# Aberrant hippocampal transmission and behavior in mice with a stargazin mutation linked to intellectual disability

**DOI:** 10.1038/s41380-022-01487-w

**Published:** 2022-03-07

**Authors:** G. L. Caldeira, A. S. Inácio, N. Beltrão, C. A. V. Barreto, M. V. Rodrigues, T. Rondão, R. Macedo, R. P. Gouveia, M. Edfawy, J. Guedes, B. Cruz, S. R. Louros, I. S. Moreira, J. Peça, A. L. Carvalho

**Affiliations:** 1 CNC-Center for Neuroscience and Cell Biology, University of Coimbra, 3004-504 Coimbra, Portugal; 2 IIIUC-Institute for Interdisciplinary Research, University of Coimbra, 3030-789 Coimbra, Portugal; 3 PhD Program in Experimental Biology and Biomedicine (PDBEB), University of Coimbra, Coimbra, Portugal; 4 Department of Life Sciences, University of Coimbra, 3000-456 Coimbra, Portugal; 5 Present Address: HEMEX AG, Liestal, Switzerland; 6 Present Address: Champalimaud Research, Champalimaud Centre for the Unknown, Lisbon, Portugal; 7 Present Address: Centre for Discovery Brain Sciences, University of Edinburgh, Edinburgh, UK

**Keywords:** Molecular biology, Biochemistry, Neuroscience, Cell biology

## Abstract

Mutations linked to neurodevelopmental disorders, such as intellectual disability (ID), are frequently found in genes that encode for proteins of the excitatory synapse. Transmembrane AMPA receptor regulatory proteins (TARPs) are AMPA receptor auxiliary proteins that regulate crucial aspects of receptor function. Here, we investigate a mutant form of the TARP family member stargazin, described in an ID patient. Molecular dynamics analyses predicted that the ID-associated stargazin variant, V143L, weakens the overall interface of the AMPAR:stargazin complex and impairs the stability of the complex. Knock-in mice harboring the V143L stargazin mutation manifest cognitive and social deficits and hippocampal synaptic transmission defects, resembling phenotypes displayed by ID patients. In the hippocampus of stargazin V143L mice, CA1 neurons show impaired spine maturation, abnormal synaptic transmission and long-term potentiation specifically in basal dendrites, and synaptic ultrastructural alterations. These data suggest a causal role for mutated stargazin in the pathogenesis of ID and unveil a new role for stargazin in the development and function of hippocampal synapses.

## Introduction

Most of the fast component of excitatory neurotransmission in the central nervous system is mediated by glutamate receptors of the α-amino-3-hydroxyl-5-methyl-4-isoxazole-propionate type (AMPAR). These receptors are associated with auxiliary proteins that regulate their traffic, gating and pharmacology, increasing receptor functional diversity in the brain [1–3]. Members of the transmembrane AMPAR regulatory protein (TARP) family are widely expressed AMPAR auxiliary subunits [4], and key modulators of AMPAR-mediated transmission. The prototypical TARP stargazin (also known as TARP γ2) was discovered in the ataxic stargazer mouse [5], which lacks synaptic AMPARs on cerebellar granule cells [6]. Stargazin interacts with both AMPA receptor subunits and synaptic PDZ-containing proteins such as postsynaptic density protein 95 (PSD95) [7] and this is required for targeting AMPAR to synapses [7–9]. TARPs, including stargazin, couple with the majority of AMPAR complexes in the brain, promote receptor trafficking to the cell surface and their synaptic targeting, and augment their functional properties [1, 2]. Not surprisingly, stargazin regulates baseline synaptic transmission and is also involved in Hebbian and homeostatic forms of synaptic plasticity that are dependent on tightly regulated AMPAR traffic [10–12].

Impaired glutamatergic synaptic transmission and plasticity have been implicated in neurodevelopmental disorders [13]. Evidence from human genetic studies suggests that copy number variation or the presence of rare point mutations in genes encoding proteins of the ionotropic glutamate receptor complex may play a role in the aetiology of these disorders [14–18]. Single nucleotide polymorphisms in the *CACNG2* gene encoding stargazin were associated with a subgroup of schizophrenia patients [19], and alterations in the DNA copy number and in the levels of stargazin mRNA were detected in the post-mortem brain of schizophrenia patients [20, 21]. Dysregulated stargazin expression was also found in the dorsolateral prefrontal cortex of patients with bipolar disorder [21], and stargazin polymorphisms were associated with the response to lithium, a frequent treatment for bipolar disorder [21, 22]. A de novo missense mutation in *CACNG2* has been identified in a non-syndromic intellectual disability (ID) patient with moderate severity [16]. Taken together, these data point to a possible link between stargazin and the pathogenesis of neurodevelopmental disorders, which has not yet been investigated. Evaluating how human mutations in the stargazin-encoding gene disrupt synaptic function and impact behavior may also provide insight into the physiological role of stargazin.

Here, we investigated the impact of the ID-associated missense V143L mutation in stargazin [16] in the molecular dynamics (MD) of the AMPAR:stargazin complex, in the cell surface diffusion of stargazin and in its ability to traffic AMPAR to the neuronal surface and to the synapse. To evaluate behavioral, neuronal morphology and functional alterations triggered by the stargazin V143L variant, we generated a knock-in (KI) mouse model to express the mutant protein. We found that stargazin V143L KI mice display alterations in cognitive and social behavior, along with altered hippocampal spine morphology, associated with synaptic ultrastructural defects. We also found disrupted synaptic transmission and plasticity and aberrant stargazin phosphorylation in stargazin V143L mutant mice.

## Results

### Intellectual disability-associated stargazin V143L mutation affects the AMPAR:stargazin complex structure

**Fig. 1:**
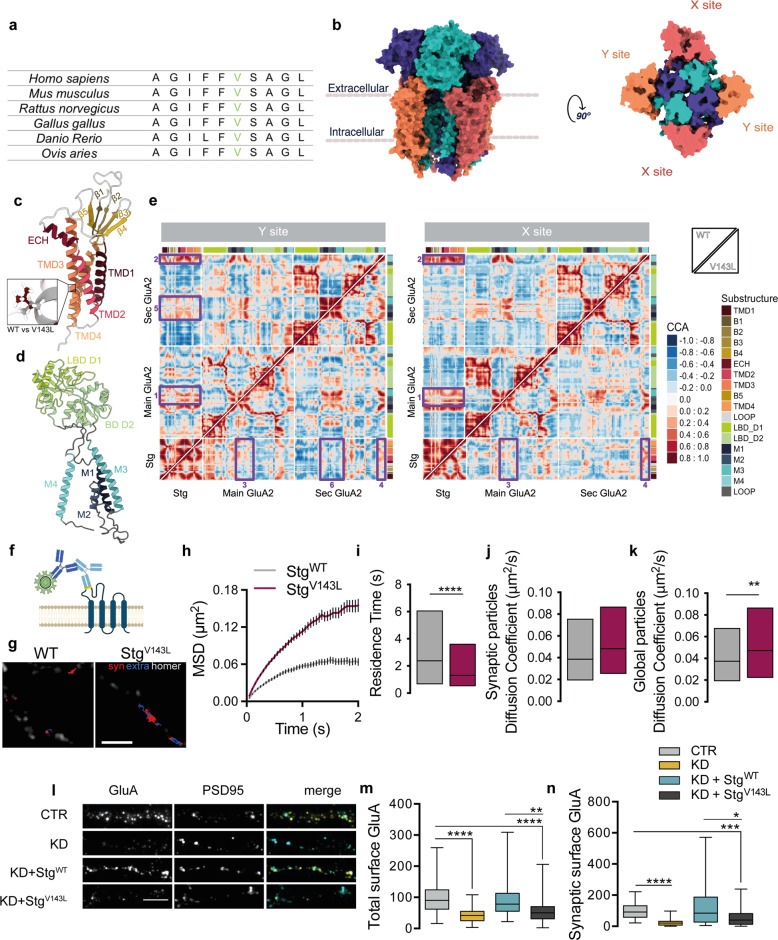
The ID-associated V143L stargazin mutation weakens the interaction between stargazin and AMPARs, presents altered surface diffusion and elicits defective AMPAR trafficking. **a** Valine 143 in stargazin is highly conserved among species. **b** Surface representation of AMPAR:stargazin complex viewed parallel to the membrane (left) and from the extracellular side (right). The extracellular view of the complex (at the membrane level) shows the two different sets of stargazin assembly points (X and Y sites) around AMPAR. Each GluA subunit is colored individually in shades of blue. Stargazin molecules are colored in orange (Y site) or brick (X site). **c** Side view of the stargazin structure shown as a cartoon with substructures labeled and colored in a spectrum of yellow/orange. Close-up shows the V143L mutation (WT – gray; ID – red). TMDs, transmembrane domains; ECH, extracellular helix. **d** Side view of a GluA2 subunit structure shown as cartoon with substructures labeled and transmembrane domains colored in a spectrum of blue and ligand-binding domain colored in green. LBD, ligand-binding domain; M1-M4, transmembrane domains. **e** Dynamical cross-correlation maps for the AMPAR:stargazin complex. Top triangle - WT complex, bottom triangle - V143L complex. Substructure annotation was added at the bottom and right of each map for easier reading. CCA goes from -1 (anticorrelated, opposite direction) to 1 (correlated, same direction). Violet boxes highlight the major differences between WT and ID dynamical CCA maps. The loops between TMD1-TMD2 and TMD3-TMD4 in stargazin are highly anticorrelated with the rest of the structure, whereas in the X site the movements are widely positively correlated. In the *Main GluA*, LBD-D1 and LBD-D2 are anticorrelated for the Y site and positively correlated for the X site. In the stargazin V143L system these differences are attenuated. In the complex containing stargazin V143L, both sites are closer to the Y site’s motions of the complex containing the WT protein. The system shows a positive correlation between M1-M2-M3 of *Main GluA* (Boxes 1) and M4 of the *Secondary GluA* (Boxes 2) with the transmembrane regions of stargazin in both X and Y sites. The introduction of the V143L stargazin mutation shifts some regions of the complex to an anticorrelated motion, especially in the M2-M3 of *Main GluA* (Boxes 3) and M4 of *Secondary GluA* (Boxes 4) (observed in both sites). In the Y site, the β1-β4 region in stargazin displays a stronger positive correlation with the LBD-D2 of the *Main GluA* when compared to the X site, which is also weakened in the mutated system. In the Y site, stargazin has a slight positive correlation with the *Secondary GluA* transmembrane domain that is lost in the mutated systems (Box 5 and 6). In the X site this correlation is never present. See also Figs. S1–S3. **f** Cortical neurons were co-transfected with Homer1C-GFP and HA-stargazin (WT stargazin – Stg^WT^, or the ID-associated variant – Stg^V143L^). Stargazin surface diffusion was evaluated using quantum dot (QD)-labeled secondary antibodies (dark blue) against anti-HA antibodies (light blue) to detect the extracellular HA epitope (yellow) in stargazin. **g** Reconstructed HA-stargazin trajectories (synaptic and perisynaptic - red, extrasynaptic - blue) and Homer1C-GFP signal (white). Scale bar represents 5 µm. **h** Stargazin mean square displacement (MSD) (±SEM) versus time plots for cells expressing WT stargazin (≥603 trajectories) or the V143L variant (≥499 trajectories), plotted for 40 time points. **i** Synaptic residence time (median ± 25–75% interquartile range) of WT and V143L stargazin. ****p < 0.0001, two-tailed Mann–Whitney test. **j**, **k** Surface diffusion coefficient of global (**j**) and synaptic (**k**; Homer1C-GFP-colocalized) single QD-stargazin particles. Median diffusion (±25–75% interquartile range) of 309 for global trajectories and 153 for synaptic trajectories. **p = 0.0054 and *p* = 0.0711, two-tailed Mann–Whitney test, respectively. A minimum of 18 cells were analyzed from *N* = 3 independent experiments. QD diffusion was followed in 600 consecutive frames of 50 ms (total of 30 s). **l**–**n** Disrupted AMPAR surface expression in the presence of the stargazin V143L variant. Low-density cortical neurons were transfected at 7–11 days in vitro (DIV) with a control plasmid (pLL-shRNA-CTR) or with pLL-shRNA-Stg, or co-transfected with pLL-shRNA-Stg and pcDNA-Stg^WT^ or pcDNA-Stg^V143L^. Total surface and synaptic levels of GluA subunits were analyzed by immunocytochemistry at DIV11-15. **l** Representative images of GluA distribution (scale bar represents 5 μm) and quantification of total (**m**) and synaptic (**n**) intensity of GluA clusters. GluA accumulation at synaptic sites was assessed by the colocalization with PSD95 clusters. **m**, **n** Clusters were quantified from at least 53 cells imaged from 6 independent experiments. Kruskall-Wallis test (*p* < 0.0001), followed by Dunn’s multiple comparison test, ***p < 0.001, ****p < 0.0001. Boxes show 25th and 75th percentiles, whiskers range from the minimum to the maximum values, and the horizontal line shows the median value.

A de novo missense mutation in the *CACNG2* gene encoding stargazin was described in a heterozygous 8 year-old male patient with moderate, non-syndromic, intellectual disability [16]. This mutation leads to substitution of valine143 by leucine (p.V143L), a residue in the third transmembrane domain of stargazin that is highly conserved among species (Fig. 1a, c), suggesting a critical role for the function of stargazin. Accordingly, the V143L substitution was predicted to be damaging using PolyPhen-2 [23], SIFT [24] and PROVEAN [25]. Importantly, this variant has not been described in databases collecting sequencing variants for the general population (Genome Aggregation Database or Exome Variant Server).

In order to characterize the effect of the V143L stargazin mutation in the structure and dynamics of the AMPAR:stargazin complex, we used molecular dynamics (MD) simulations. To the best of our knowledge, there are no MD studies available regarding AMPAR:TARP complexes. We applied MD algorithms to predict in silico how the AMPAR:stargazin system responds to a particular perturbation. To this purpose we used homology modeling to construct both the WT and V143L models of the AMPAR:stargazin complex (Fig. 1b–d), based on one of the described cryo-EM structures for the complex [26]. AMPARs are composed of two dimers comprised of A/D and B/C subunits, providing high conformational flexibility [27, 28]. AMPAR:TARPs shows variable stoichiometry with an apparent maximum of four TARPs that can be broken down into two groups: TARPs binding X sites (common interfaces with AMPAR subunits A/B or C/D) and Y sites (involving subunits A/D or B/C) [28] (Fig. 1b). We will herein refer to the GluA structure exhibiting the highest contact surface with the coupled TARP as *Main GluA* and *Secondary GluA* to the other one. We analyzed the effect of the stargazin V143L mutation at both sites considering different metrics on macromolecular rearrangements such as cross-correlation analysis (CCA) and root mean square deviations (RMSD), and at the interfacial level (solvent accessible surface area - SASA). Free binding energy (ΔG_total_) calculations were also performed to assess the mutant effect on the complex binding stability.

Figure 1e reveals the network of correlated/anticorrelated (same/opposite direction) motions between different regions of the AMPAR:stargazin complex structure, which informs on the impact of the stargazin V143L mutation in the overall structural conformation of the macromolecular complex. Overall CCA results indicate that the X site is more prone to conformational rearrangements introduced by the V143L mutation in stargazin than the Y site. RMSD was used to assess protein conformational changes between different time points in the trajectory, and their distribution is shown in Figs. S1 and S2 for all monomers at both sites, in the WT and mutated complexes. V143L stargazin-containing mutated complexes show lower density for lower values than WT complexes, demonstrating a higher flexibility. Furthermore, ΔΔSASA values (ΔSASA_V143L_ – ΔSASA_WT_) tend to be negative, which means that the interface area is larger in the WT complex when compared to V143L. This is particularly relevant for transmembrane domain 1 (M1) of *Main GluA* and the transmembrane domains (TMD) 3 and 4 of stargazin. The X site shows the greatest differences between mutant and WT stargazin, suggesting that the stargazin V143L mutation especially hinders AMPAR:stargazin interaction at this site (Fig. S3). Last, ΔΔG_total_ = ΔG_V143L_ – ΔG_WT_ = 6.20 ± 0.55 kcal/mol (*p* < 0.0001), which shows that V143L leads to a decrease in the binding affinity between AMPAR and stargazin. This difference is particularly relevant for the X site (Table 1). Taken together, the prediction from the MD analysis is that the V143L mutation in stargazin weakens the interaction of stargazin with the AMPAR complex, particularly in the X interaction site.

**Table 1:** Free binding energy values for AMPAR:stargazin complexes containing WT or V143L stargazin.

Site	ΔG_WT_ (kcal/mol)	ΔG_V143L_ (kcal/mol)	ΔΔG (ΔG_V143L_–ΔG_WT_)	*p* value
Both	−42.98 ± 0.39	−36.78 ± 0.40	6.20 ± 0.55	<0.0001
X	−40.02 ± 0.28	−30.78 ± 0.53	9.24 ± 0.60	<0.0001
Y	−42.02 ± 0.42	−41.78 ± 0.51	0.24 ± 0.66	0.212

ΔΔG values were obtained from ΔG_V143L_ – ΔG_WT_, and are presented in kcal/mol. Wilcoxon test was used to calculate *p* value.

### The V143L mutation affects the trafficking properties of stargazin

Stargazin plays a role in AMPAR trafficking through the early compartments of the biosynthetic pathway [29], and mediates complexed AMPAR trafficking to the cell membrane, their synaptic stabilization [7, 30] and surface diffusion trapping [8, 31] through binding to PSD95. Given the described roles, we explored the potential effect of the V143L mutation on stargazin’s cell surface diffusion properties. Low-density cortical neurons were co-transfected with plasmids encoding Homer-GFP, for synapse identification, and HA-tagged WT stargazin (Stg^WT^), or the V143L stargazin variant (Stg^V143L^). We monitored stargazin diffusion by single nanoparticle imaging of HA-stargazin using quantum dots (QDs; Fig. 1f–k). Stargazin V143L particles displayed increased mean square displacement (MSD; Fig. 1h), decreased synaptic residence time (Fig. 1i) and higher diffusion coefficient than Stg^WT^ (Fig. 1k), suggesting that the V143L mutation renders stargazin more mobile in the plasma membrane.

The ID-associated mutation is located in the third transmembrane domain of stargazin (Fig. 1c), which was shown to be involved in the interaction with AMPAR subunits [32, 33]. Our molecular dynamics analyses indicate that this mutation weakens the interaction of stargazin with the AMPAR complex, in particular in the X site (Fig. 1e and Table 1). We thus hypothesized that stargazin V143L may be defective in trafficking AMPAR to the cell surface and to the synapse. To test this possibility, we used a molecular replacement strategy in which we silenced endogenous stargazin expression in cultured cortical neurons with a specific shRNA [11] and re-introduced either WT stargazin or the V143L variant. We assessed the effect of stargazin depletion and of the expression of the stargazin V143L variant in AMPAR trafficking and synaptic stabilization in cultured neurons (Fig. 1l–n). Cell surface and synaptic expression levels of AMPAR were evaluated by immunolabeling GluA AMPAR subunits using an antibody specific for their extracellular N-terminal region (Fig. 1l). As previously described [11], stargazin silencing led to a decrease on the cell surface (Fig. 1l, m) and synaptic levels of AMPAR (Fig. 1l, n). AMPAR clusters were considered synaptic when colocalizing with PSD95, whose expression was not affected by stargazin silencing (data not shown). In cells co-transfected with stargazin shRNA and WT shRNA-refractory stargazin (KD + Stg^WT^), total and synaptic surface levels of GluA were rescued to basal levels. Critically, neuronal transfection of shRNA-refractory stargazin V143L mutant (KD + Stg^V143L^) led to a failure in mediating normal AMPAR traffic to the cell surface (Fig. 1l, m) and to the synapse (Fig. 1l, n), showing that the ID-associated mutation impairs stargazin’s role in AMPAR trafficking.

### Genetically engineered mice with the stargazin V143L mutation show altered cognitive and social behavior

In order to study the effects of the ID-associated stargazin mutation in vivo, we generated a knock-in (KI) mouse line in which the human mutation was introduced in the mouse *Cacng2* gene. Using the gene targeting strategy we targeted the *Cacng2* gene to modify the nucleotide in the third exon which was found to be mutated in the ID patient [16] (Fig. S4a). Confirmation of the mutation was performed by Sanger sequencing (Fig. S4b). Heterozygous and homozygous KI mice were viable, did not display gross abnormalities, and did not show spontaneous seizures. To determine whether expression of the stargazin V143L variant affects gross brain morphology, we performed Nissl staining in brain coronal slices and compared sections from WT and homozygous stargazin V143L KI (KI^VL/VL^) mice. As shown in Fig. S4c, no apparent macroscopic defects were visible in the brain of stargazin KI^VL/VL^ animals, suggesting that overall brain morphology is not affected by the stargazin mutation. Moreover, the structural organization of the hippocampus and cortical lamination were preserved (Figs. S4c, f, g).

To assess whether the V143L stargazin mutation affects stargazin protein levels and distribution across the brain, we performed immunolabeling of stargazin in brain coronal and sagittal slices. Stargazin is broadly expressed throughout the mouse brain with high expression levels in the cerebral cortex, hippocampus, and cerebellum [4]. Within the hippocampus, stargazin immunoreactivity was most intense in the *stratum oriens* of the CA1, CA2 and C3 regions, the *stratum lacunosum moleculare* of the CA1 and CA2 regions, and particularly in the *subiculum* (Figs. S4d–f). Stargazin immunoreactivity was similar in all genotypes (Figs. S4d–g), indicating that the expression of mutated stargazin does not affect the protein brain-wide distribution and total expression levels. This was also confirmed by western blot analyses, using total lysates from the whole brain, cortex and hippocampus (Figs. S4h–j). The expression levels of stargazin and other TARPs were also assessed by qPCR and no changes were detected in the cortex and hippocampus of KI mice, compared to WT littermates (Fig. S4k).

Since the V143L stargazin mutation was found in an ID patient [16], we asked whether stargazin V143L KI mice display alterations in motor function, anxiety-like behavior, cognitive and/or social performance that correlate with ID-like symptomatology. We began by assessing motor behavior in the open field test (Fig. S5a) and found that, whereas male stargazin KI^+/VL^ and KI^VL/VL^ mice showed comparable distance traveled and instant speed to WT male mice (Figs. S5c, e), female stargazin KI^VL/VL^ mice traveled longer distances, and stargazin KI^+/VL^ female mice showed higher instant speed than WT female mice (Figs. S5b, d), suggesting hyperactivity in female stargazin V143L KI animals. However, stargazin V143L KI mice did not display anxiety-like behaviors either in the open field (Fig. S5f) or in the elevated plus maze (Figs. S5g, h) tests, nor did they show depressive-like behavior in the forced swimming test (Figs. S5i, j). Heterozygous stargazin V143L KI animals failed to alternate above chance level in the T-maze spontaneous alternation test for working memory (Figs. S5k, l).

**Fig. 2:**
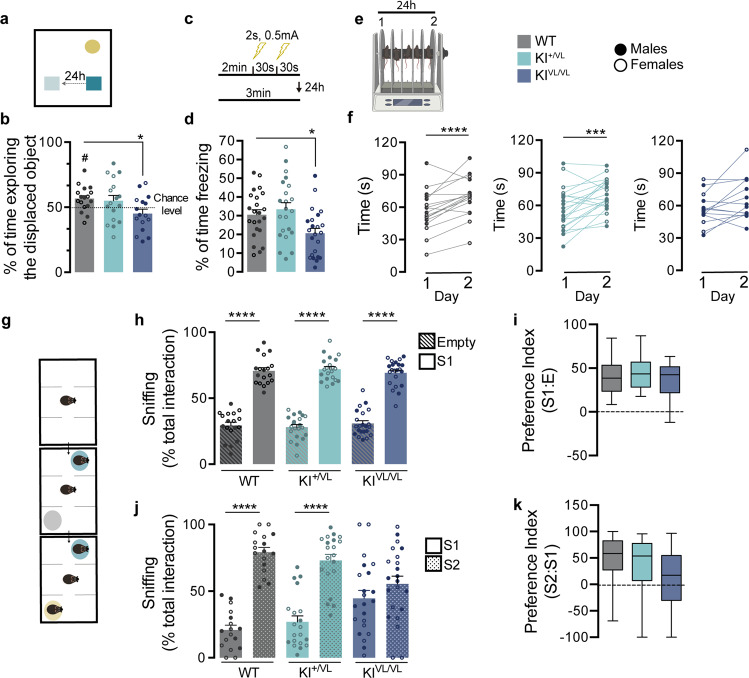
Stargazin V143L KI mice show cognitive and social deficits. **a**, **b** When subjected to the object displacement recognition test, homozygous stargazin V143L (KI^VL/VL^) mice spent less time exploring the displaced object when compared to their WT counterparts, and did not have preference for the displaced object. Data are presented as mean ± SEM. One-way ANOVA (*p* = 0.0412) followed by Dunnet’s multiple comparison test, *p < 0.05; one-sample *t* test to the value of 50%, #*p* = 0.013. *N* ≥ 16 (males and females) for all genotypes. **c**, **d** Homozygous stargazin V143L knock-in mice presented significantly less freezing behavior than WT counterparts in the contextual fear conditioning test. Data are presented as mean ± SEM. One-way ANOVA (*p* = 0.0076) followed by Dunnett’s multiple comparison test, *p < 0.05. *N* ≥ 22 (males and females) for all genotypes. **e** Motor function and learning were evaluated using the rotarod test. **f** The average time spent on the rotarod on the first day did not significantly vary between genotypes (one-way ANOVA, *p* = 0.9990). Both WT and heterozygous stargazin V143L (KI^+/VL^) animals performed significantly better in the second day, whereas stargazin KI^VL/VL^ mice failed to show motor learning. Ratio paired *t* test, ****p < 0.0001 for WT mice, ***p = 0.0009 for KI^+/VL^ mice and *p* = 0.0522 for KI^VL/VL^ mice. *N* ≥ 14 for all genotypes (males and females). **g** Mice were submitted to the three‐chamber social interaction paradigm. The time spent approaching the cages, with and without the stranger stimulus mouse, was evaluated for 10 and 5 min, respectively. **h**, **i** All animals displayed social preference, but (**j**, **k**) stargazin KI^VL/VL^ mice showed no preference for a new stranger mouse in the arena, unlike WT and heterozygous stargazin V143L mice. Data are presented as mean ± SEM (**h**, **j**) and median ± IQR (whiskers represent minimum and maximum values) (**i**, **k**). Two-way ANOVA [(**h**) *p* < 0.0001 (S1 vs empty), *p* > 0.9999 (genotype), *p* = 0.4107 (interaction); (**j**) *p* < 0.0001 (S1 vs S2), *p* > 0.9999 (genotype), *p* < 0.0001 (interaction)] followed by Sidak’s multiple comparison test, ****p < 0.0001. *N* ≥ 17 (males and females) for all genotypes. See also Fig. S5.

In an object displacement test for spatial memory evaluation, while WT animals preferred to spend time engaging with the displaced object, neither stargazin KI^+/VL^ nor KI^VL/VL^ mice showed this preference, and stargazin KI^VL/VL^ mice spent significantly less time exploring the object that was moved when compared to WT animals (Fig. 2a, b). Furthermore, in the contextual fear conditioning test for associative memory, stargazin KI^VL/VL^ mice presented less freezing behavior than WT animals (Fig. 2c, d). These observations suggest that the V143L mutation in stargazin elicits learning and memory impairments. Given the high expression of stargazin in the cerebellum [4], we assessed motor learning of stargazin V143L KI animals in the rotarod test (Fig. 2e, f). No significant motor abnormalities were displayed by mutant mice in the rotarod test in the first day of the test, but whereas WT and stargazin KI^+/VL^ mice improved their performance the second day they were placed in the apparatus, stargazin KI^VL/VL^ mice failed to do so, suggesting an impairment in motor learning (Fig. 2f). Typically, ID patients display deficits in several social skills, including the will/ability to socially engage with other people. To determine whether stargazin V143L KI mice display social interaction deficits, we tested these animals in the three-chamber test. Stargazin V143L KI mice showed preference for a conspecific (Stranger 1 - S1) over an empty cage (E), similarly to WT mice (Fig. 2g–i). However, in the presence of a novel social partner (Stranger 2 - S2), contrarily to WT and stargazin KI^+/VL^ mice, stargazin KI^VL/VL^ mice did not prefer to interact with the unfamiliar animal (Fig. 2g, j, k). This result suggests a possible deficit in social recognition and/or alterations in the motivation for social novelty. The innate social behavior of nest building was not perturbed in stargazin V143L KI mice (Figs. S5m, n). Together, our results show that the ID-associated mutation in stargazin elicits cognitive and social deficits reminiscent of ID-like symptoms.

### Stargazin V143L mutant mice exhibit early hippocampal synaptic transmission defects

**Fig. 3:**
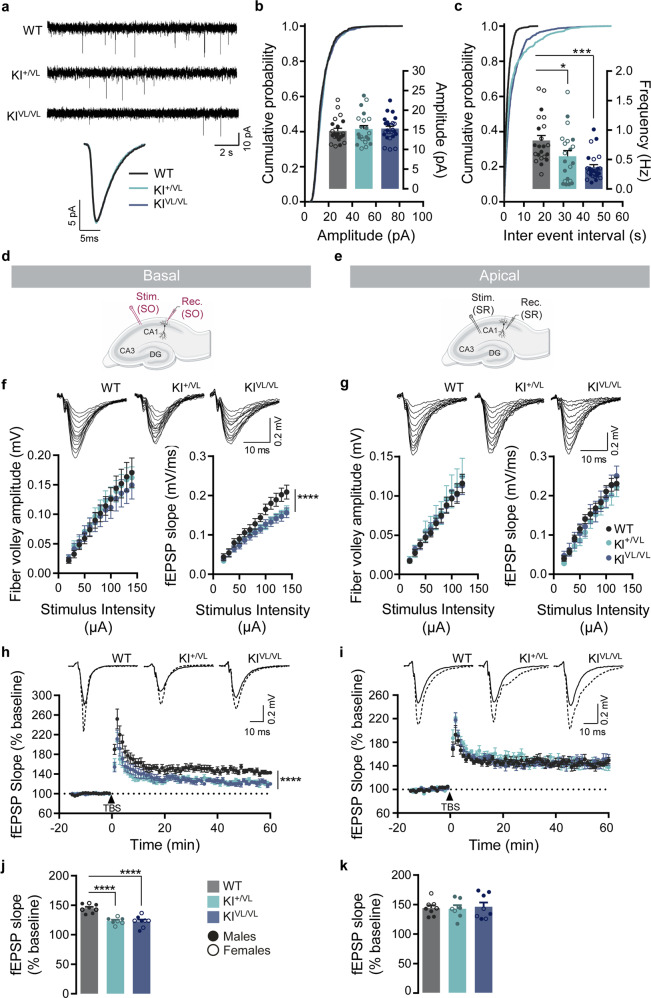
Stargazin V143L KI mice present decreased excitatory synaptic transmission and plasticity in CA1 pyramidal neurons. **a** Representative traces of mEPSCs recordings and single average event of CA1 pyramidal neurons in acute hippocampal slices from WT, stargazin KI^+/VL^ and stargazin KI^VL/VL^ mice. **b** Cumulative probability distribution and average mEPSCs amplitude and (**c**) frequency plots, showing a reduction in frequency but not amplitude of mEPSCs in stargazin V143L KI mice (P15-P20). Data are presented as mean ± SEM. Kruskal–Wallis (amplitude: *p* = 0.9803; frequency: *p* = 0.0003) followed by Dunn’s multiple comparisons test, *p < 0.05, ***p < 0.001. *n* = 22 cells, *N* = 10 animals (4 males and 6 females) for WT mice; *n* = 21 cells, *N* = 8 animals (4 males and 4 females) for KI^+/VL^ mice; *n* = 25 cells, *N* = 6 animals (3 males and 3 females) for KI^VL/VL^ mice. **d** Schematic representation of electrodes placement for evoked fEPSPs in CA1 basal and (**e**) apical dendritic synapses. **f** Representative traces of evoked fEPSPs at CA1 basal and (**g**) apical synapses on hippocampal slices from P15-P20 WT and stargazin V143L KI mice, upon stimulation of axons in the *stratum oriens* (SO) or *stratum radiatum* (SR), respectively. Plots show the mean ± SEM of fiber volley amplitude and fEPSP slopes. **f** No changes were observed in the fiber volley amplitude between genotypes when recording from basal dendritic synapses [two-way repeated measures ANOVA, *p* = 0.7473 (interaction), *p* = 0.8996 (genotype), *p* < 0.0001 stimulus intensity)]. However, fEPSP responses were decreased [two-way repeated measures ANOVA, ****p < 0.0001 (interaction), *p* = 0.0957 (genotype), *p* < 0.0001 (stimulus intensity)]. *n* = 9 slices, *N* = 5 animals (2 males and 3 females) for WT mice, *n* = 12 slices, *N* = 6 animals (3 males and 3 females) for stargazin KI^+/VL^ mice, *n* = 15 slices, *N* = 5 animals (3 males and 2 females) for stargazin KI^VL/VL^ mice. **g** Input-output curves recorded in apical dendritic synapses were similar for all genotypes, indicating that evoked basal transmission in this synapse is not impaired in stargazin V143L KI mice [two-way repeated measures ANOVA; fiber volley: *p* = 0.9996 (interaction), *p* = 0.8860 (genotype), *p* < 0.0001 (stimulus intensity); fEPSP slope: *p* = 0.7585 (interaction), *p* = 0.7391 (genotype), *p* < 0.0001 (stimulus intensity)]. *n* = 17 slices, *N* = 10 animals (5 males and 5 females) for WT mice; *n* = 11 slices, *N* = 8 animals (4 males and 4 females) for stargazin KI^+/VL^ mice; *n* = 15 slices, *N* = 10 animals (6 males and 4 females) for stargazin KI^VL/VL^ mice. **h** LTP induced by theta burst stimulation (TBS) was impaired at CA1 basal synapses [two-way repeated measures ANOVA, *p* = 0.3715 (interaction), *p* = 0.7383 (genotype), *p* < 0.0001 (time)], whereas in (**i**) CA1 apical synapses LTP was comparable between genotypes [two-way repeated measures ANOVA, *****p < 0.0001 (interaction), *p* = 0.0030 (genotype), *p* < 0.0001 (time)]. Insets show representative traces of evoked fEPSPs before (solid lines) and after (dashed lines) LTP induction. Data are presented as mean ± SEM. For basal synapses *n* = 8 slices, *N* = 5 animals (2 males and 3 females) for WT mice; *n* = 7 slices, *N* = 4 animals (1 male and 3 females) for stargazin KI^+/VL^ mice; *n* = 8 slices, *N* = 4 animals (2 males and 2 females) for stargazin KI^VL/VL^ mice. For apical synapses *n* = 8 slices, *N* = 6 animals (3 males and 3 females) for WT mice; *n* = 7 slices, *N* = 5 animals (3 males and 2 females) for stargazin KI^+/VL^ mice; *n* = 8 slices, *N* = 5 animals (3 males and 2 females) for stargazin KI^VL/VL^ mice. **j** Average fEPSP slope in the last 10 min of the recording post LTP-induction for basal and (**k**) apical CA1 dendritic synapses. Data are presented as mean ± SEM. One-way ANOVA (*p* < 0.0001 for basal synapses and *p* = 0.9267 for apical synapses) followed by Dunnett’s multiple comparisons test, ****p < 0.0001.

To assess whether the decrease in surface AMPA receptor levels observed in vitro in neurons expressing stargazin V143L has an impact in glutamatergic transmission in vivo, we performed whole-cell patch-clamp recordings in CA1 pyramidal neurons from acute hippocampal slices of P15-P20 stargazin V143L KI mice, to measure AMPA receptor-mediated miniature excitatory post-synaptic currents (mEPSCs). We found that the frequency of mEPSCs events was significantly decreased in neurons from stargazin KI^+/VL^ and KI^VL/VL^ mice compared to WT littermates (Fig. 3a, c). Interestingly, no changes in the amplitude of mEPSCs (Fig. 3a, b) or in the kinetics of these events (Fig. 3a) were observed.

We next investigated the consequences of the ID-associated stargazin mutation in hippocampal functional connectivity and synaptic plasticity by recording field excitatory post-synaptic potentials (fEPSPs) in CA1 basal and apical dendrites while stimulating the Schaffer collateral fibers in the *stratum oriens* (Fig. 3d) or in the *stratum radiatum* (Fig. 3e), respectively. First, we tested the impact of the V143L stargazin mutation on synaptic transmission and found decreased fEPSP responses when recording from the *stratum oriens* (Fig. 3f) but not when recording from the *stratum radiatum* (Fig. 3g). This suggests that the connectivity to CA1 post-synaptic sites is impaired in basal dendritic synapses but preserved in apical dendritic synapses in the hippocampus CA1 region of stargazin V143L KI animals. No significant alterations were found in the fiber volley amplitude between genotypes, indicating that there are no gross presynaptic impairments in stargazin V143L KI mice at these synapses (Fig. 3f, g). Indeed, when paired-pulse facilitation, a short-term strengthening of synaptic transmission, was assessed no overt alterations were observed (Figs. S6), further supporting that the presynaptic function is intact in these hippocampal pathways in stargazin V143L KI mice. Finally, we induced long-term potentiation (LTP) in acute hippocampal slices using a theta burst stimulation protocol. Stargazin V143L KI mice showed decreased LTP recorded in basal CA1 synapses (Fig. 3h, j), but normal LTP at apical CA1 synapses (Fig. 3i, k). Altogether, these data indicate that synaptic connectivity and theta burst-induced long-term synaptic potentiation are specifically impaired in synapses in basal dendrites in the CA1 region of the hippocampus in stargazin V143L KI mice.

### Stargazin V143L mutant mice have reduced mature spine density on basal dendrites of CA1 hippocampal neurons

Changes in dendritic branch complexity and length, and spine density, volume and shape have been described in the brains of patients with neuropsychiatric disorders (reviewed in [34]). To understand if the stargazin V143L mutation impacts neuronal morphology, we performed Sholl analysis of CA1 pyramidal neurons of stargazin V143L KI mice. For that, we intravenously injected AAV9.hSyn.GFP in the mice to achieve sparse, Golgi-like, labeling of neurons and outline the morphology of dendrites and dendritic spines (Figs. S7a, b). Our results show that the stargazin V143L mutation has no impact on dendritic arbor complexity (Figs. S7c, d) or in the total dendritic length of basal and apical dendrites (Fig. S7e).

**Fig. 4:**
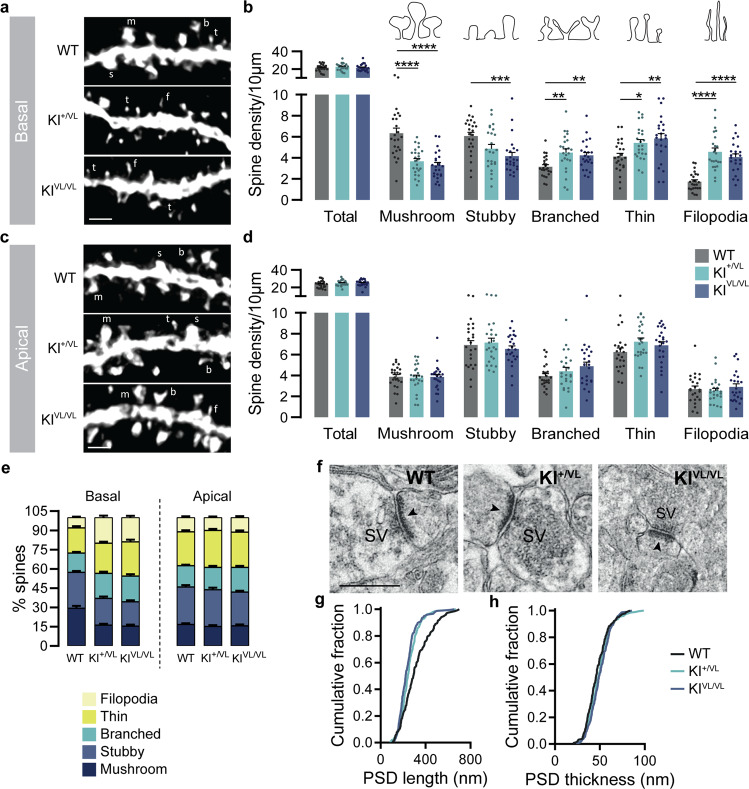
Stargazin V143L KI mice show alterations in hippocampal spine morphology and PSD ultrastructure. **a**–**e** Although no changes were observed in the total density of spines in either basal or apical dendrites in CA1 neurons, (**a**, **b**) Stargazin V143L KI animals presented a decrease in mushroom and stubby spines and an increase in branched, thin and filopodia spines in basal dendrites, indicating a defect in spine maturation. Data are presented as mean ± SEM (**b**, **d**) or as percentage of the total number of spines (**e**). Two-way repeated measures ANOVA [basal dendritic spines: *p* < 0.0001 (interaction), *p* = 0.3924 (genotype), *p* < 0.0001 (spine morphology); apical dendritic spines: *p* = 0.3172 (interaction), *p* = 0.3006 (genotype), *p* < 0.0001 (spine morphology)] followed by Dunnet’s multiple comparisons test, *p < 0.05, **p < 0.01, ***p < 0.001, ****p < 0.0001. *n* = 24 branches, *N* = 3 animals for all genotypes. m, mushroom; s, stubby; b, branched; t, thin; f, filopodia. Scale bar represents 1 μm. **f** Representative electron transmission microscopy images of hippocampal synapses from WT, stargazin KI^+/VL^ and stargazin KI^VL/VL^ animals. The arrows indicate the post-synaptic densities. SV, synaptic vesicles. Scale bar represents 500 nm. **g** Cumulative frequency distribution of post-synaptic density length and (**h**) thickness of hippocampal PSDs from WT, stargazin KI^+/VL^ and stargazin KI^VL/VL^ animals (*n* = 153 PSDs, *N* = 2 animals for WT mice; *n* = 203 PSDs, *N* = 2 animals for KI^+/VL^ mice, *n* = 236 cells, *N* = 2 animals for KI^VL/VL^ mice). See also Fig. S8.

Next, we evaluated the effects of the stargazin V143L mutation on dendritic spine density and morphology in the hippocampus of stargazin V143L KI mice. While no differences in total dendritic spine density were observed, there was a significant decrease in the density of mature spines, namely mushroom and stubby spines, on basal dendrites of CA1 pyramidal neurons from stargazin KI^+/VL^ and KI^VL/VL^ mice when compared to WT littermates (Fig. 4a, b). Additionally, stargazin KI^+/VL^ and KI^VL/VL^ mice displayed increased density of branched spines and of immature spines (thin and filopodia) on basal dendrites (Fig. 4a, b). In contrast, no changes in spine density or morphology were found on apical dendrites of CA1 pyramidal neurons of stargazin V143L KI mice (Fig. 4c, d). Overall, there was a significant decrease in the percentage of mature spines on basal dendrites of stargazin V143L KI mice neurons, whereas no changes were observed on the morphology of spines on apical dendrites (Fig. 4e). These data show that the stargazin V143L mutation specifically results in spine dysmorphogenesis on basal dendrites. Interestingly, in the CA1 region stargazin was more expressed in the *stratum oriens*, where the basal dendrites are located, when compared to the *stratum radiatum*, where apical dendrites are placed (Fig. S4e). This differential pattern of stargazin expression within the CA1 region correlates with the pronounced effects in spine maturation observed on basal dendrites.

To further explore the alterations in spine morphology, we performed ultrastructural analysis of the post-synaptic density (PSD) in hippocampal spines from WT and stargazin V143L KI mice using electron microscopy. Our analysis uncovered a decrease in the length of PSDs from stargazin KI^+/VL^ and KI^VL/VL^ mice (Fig. 4f, g), as well as a tendency for an increase in the thickness of PSDs from stargazin KI^VL/VL^ mice when compared with WT littermates (Fig. 4f, h), highlighting potential alterations in post-synaptic structure and composition. Moreover, the total levels of PSD95 in the hippocampus of stargazin V143L KI mice were significantly reduced compared to WT littermates (Figs. S8a, b). PSD95 has an important role in silent synapse maturation [35] and PSDs with smaller size and decreased PSD95 content are less stable [36]. The decreased PSD95 levels further support an impairment in spine maturation in the hippocampus of stargazin V143L KI mice. We analyzed the effects of the stargazin V143L mutation in the ultrastructure of PSDs in the cortex (Figs. S8c–e), and also observed a decrease in the length of PSDs of stargazin KI^VL/VL^ mice (Figs. S8c, d). However, in contrast to what was observed in the hippocampus, there was a decrease in the thickness of PSDs from stargazin KI^+/VL^ mice (Figs. S8c, e). No changes were observed in cortical PSDs total levels of PSD95 (Figs. S8f, g).

Together, these data reveal that the stargazin V143L mutation leads to an increase in the density of spines with immature morphology and to ultrastructural changes in post-synaptic compartments, indicating a general spine immaturity state in certain hippocampal subregions of stargazin V143L KI mice. Combined with our functional characterization showing decreased frequency of mEPSC events in CA1 neurons from stargazin mutant mice, as well as decreased connectivity and long-term plasticity in CA1 basal synapses, this strongly suggests that the stargazin V143L mutation perturbs spine maturation and diminishes functional synaptic contacts and plasticity in specific hippocampal subcircuits.

### Stargazin phosphorylation and interaction with GluA1 are decreased in stargazin V143L mutant mice

**Fig. 5:**
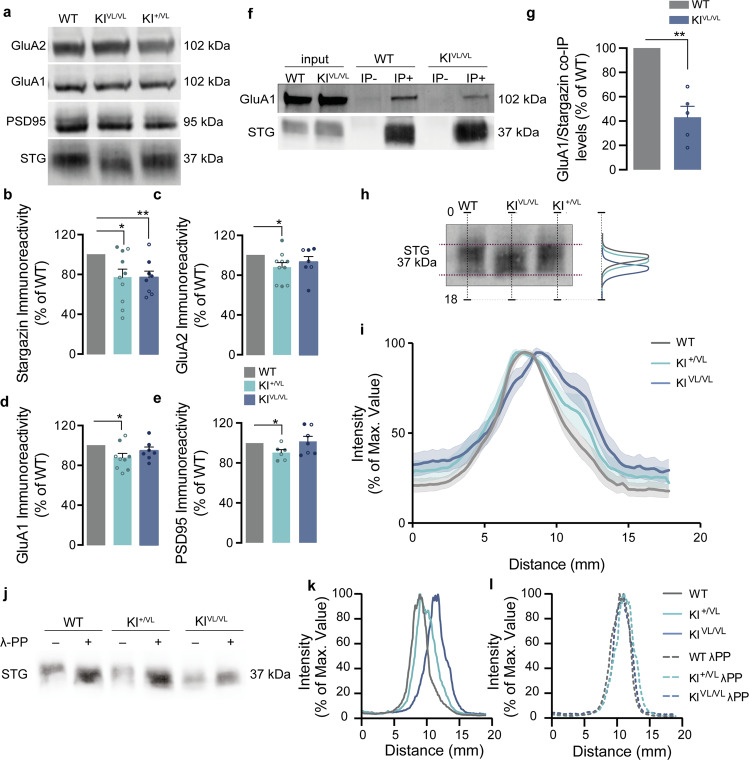
Stargazin V143L expression modifies the composition of PSDs. **a**, **b** Western blot analysis of cortical PSDs (see also Fig. S8h) showed that heterozygous stargazin KI^+/VL^ animals and homozygous stargazin KI^VL/VL^ mice have decreased synaptic levels of stargazin (*p = 0.0217 for KI^+/VL^ and **p = 0.0084 for KI^VL/VL^ animals). **a**, **c** GluA2 (*p = 0.0324 for KI^+/VL^ and *p* = 0.2709 for KI^VL/VL^ animals), (**a**, **d**) GluA1 (*p = 0.021 for KI^+/VL^ and *p* = 0.2016 KI^VL/VL^ animals) and (**a**, **e**) PSD95 (*p = 0.025 for KI^+/VL^ and *p* = 0.7638 for KI^VL/VL^ animals) levels were also reduced in the PSDs of stargazin KI^+/VL^ animals. Data were normalized for WT values and are presented as mean ± SEM. One-sample *t* test to the value of 100%. *N* ≥ 6 for all conditions. **f** Immunoprecipitation of stargazin from the cortices of WT and stargazin KI^VL/VL^ animals. **g** Co-immunoprecipitation of GluA1 with stargazin was significantly reduced in the KI^VL/VL^ cortices. Data were normalized for immunoprecipitated stargazin in each respective condition. Data normalized to WT values and presented as mean ± SEM. One-sample *t* test to the value of 100%, ***p = 0.0032, *N* = 5 for all conditions. **h** The SDS-PAGE migration pattern of stargazin from WT, stargazin KI^+/VL^ and KI^VL/VL^ mouse cortical PSD extracts was analyzed by quantifying the distribution of the intensity of the bands along the length of the lane. **i** In PSDs isolated from stargazin KI^VL/VL^ animals, stargazin migrated faster in the SDS-PAGE. Data were normalized for the average maximum intensity of WT stargazin for each western blot membrane and are presented as mean ± SEM, *N* = 9. **j** Representative images from stargazin labeling profile in cortical PSD samples isolated from WT, stargazin KI^+/VL^ and KI^VL/VL^ samples non-treated (**k**) or treated (**l**) with λ-Phosphatase. λ-Phosphatase treatment of isolated cortical PSDs induced a shift in the apparent molecular weight of stargazin in WT and stargazin KI^+/VL^ PSD samples, but not in stargazin KI^VL/VL^ PSD samples, suggesting deficient phosphorylation of the stargazin V143L protein variant detectable in homozygous stargazin V143L KI animals.

Finally, we determined whether the dendritic spines immaturity (Fig. 4a, b, e) and the decreased PSD length (Fig. 4f, g and S8c, d) found in the brain of stargazin V143L KI animals are accompanied by altered composition of the PSDs. We isolated PSDs from the cerebral cortex of WT, stargazin KI^+/VL^ and KI^VL/VL^ littermate mice (Fig. S8h) and quantified their content in stargazin, GluA1 and GluA2 AMPAR subunits, as well as PSD95 (Fig. 5a–e). Stargazin expression was decreased in the PSDs of stargazin KI^+/VL^ and KI^VL/VL^ mice compared to WT mice (Fig. 5a, b, despite not significantly changed total expression levels of stargazin in mutant mice - Fig. S4j), and heterozygous stargazin V143L mice showed decreased levels of GluA1, GluA2 and PSD95 at the PSD (Fig. 5a, c–e). In agreement with our molecular dynamics analyses (Fig. 1e), immunoprecipitation of stargazin V143L from the cerebral cortex of stargazin KI^VL/VL^ mice showed decreased co-immunoprecipitation of GluA1, compared with stargazin immunoprecipitated from the cortex of WT littermate mice (Fig. 5f, g), indicating that the V143L mutation in stargazin impairs its interaction with AMPAR subunits in vivo.

The function of stargazin is regulated by the phosphorylation of serine residues in the cytoplasmic C-terminal tail of the protein [12], and these phosphorylation events regulate stargazin interaction with membrane lipids [37], its binding to PSD95 [9, 38], and the diffusional trapping of AMPARs at synaptic sites [31]. The migration pattern of stargazin in denaturing SDS-PAGE conditions correlates with the phosphorylation state of the protein, with phosphorylated stargazin showing slower migration in SDS-PAGE [11, 12]. We noticed that, in PSDs isolated from the cortex of stargazin KI^VL/VL^ mice, stargazin showed faster migration compared to PSDs isolated from WT mice, whereas an intermediate migration pattern was detected in PSDs isolated from heterozygous stargazin V143L mice (Fig. 5h, i). To test for altered phosphorylation of mutant stargazin, we treated cortical extracts with λ-phosphatase before PSD purification and found that stargazin bands in PSDs isolated from the cortex of WT or heterozygous stargazin KI^+/VL^ mice shifted to a lower apparent molecular weight, putatively corresponding to the unphosphorylated form of the protein [11, 12] and coincident with the stargazin band in PSDs isolated from untreated cortical extracts from stargazin KI^VL/VL^ mice (Fig. 5j–l). In fact, the gel mobility of the stargazin band in PSDs isolated from stargazin KI^VL/VL^ mice was unchanged by λ-phosphatase treatment (Fig. 5j–l), indicating that the protein is in a dephosphorylated form. These findings are consistent with decreased phosphorylation of V143L stargazin.

## Discussion

In this study, we employed molecular dynamics analyses, in vitro and in vivo models to study how an ID-associated mutation in the third transmembrane domain of stargazin impacts the AMPAR:stargazin complex, hippocampal synaptic architecture, synapse function and behavior. Our data suggest that the V143L mutation in stargazin critically affects stargazin interaction with the AMPAR complex, leads to decreased stargazin phosphorylation, decreases AMPAR-mediated synaptic transmission, and contributes to spine immaturity in CA1 hippocampal neurons. A striking aspect of our study is that it reveals not only the pathogenic effect of a mutant form of stargazin associated with disease, but also unveils critical roles for stargazin in regulating synapse structure and function in the hippocampus and in shaping cognitive and social behavior.

Structural analyses highlight that the main interface between AMPAR:stargazin is in the TMD of the complex. This interaction is mainly mediated by TMD3 and TMD4 in stargazin, M1 and M2 in *Main GluA*, and M4 from *Secondary GluA*. Our analysis showed that the V143L mutation impairs the correlated motion between transmembrane regions of stargazin and GluA, especially at M4 of *Secondary GluA*.

The free-energy value is higher for the AMPAR:TARP complex containing stargazin V143L (by ~14% on the whole complex), particularly at the X site (~23% difference, Table 1). Although the theoretical free binding energy binding values for the AMPAR:TARP complex do not consider entropic effects, and assume a stoichiometry of 4 TARPs per AMPAR tetramer, and homomeric AMPAR binding exclusively stargazin, which could be variable in the complex, they still allow us to confidently rank the stability of the different complexes. The higher value determined for the V143L system demonstrates the negative effect of this single point mutation on the overall stability of the protein–protein complex, particularly at the X-site. These predictions were assessed experimentally and are in agreement with the decreased co-immunoprecipitation of GluA1 with the ID-associated variant.

To date, the physiological roles of TARPs have been studied using knock-out mice for the different TARPs, alone or in combination (reviewed in [2]). These analyses have provided crucial insight into partially overlapping although non-redundant functions for different TARPs but are hindered by possible compensatory effects that may arise in the absence of the endogenous proteins. Examining knock-in mouse models expressing mutant forms of stargazin associated with disease has the double advantage of informing on the endogenous role of stargazin, by analyzing the effects of loss of function mutant variants which are still expressed, and on possible pathogenic mechanisms elicited by human stargazin mutations. In this study, we have found that the V143L mutation triggers a striking decrease in the frequency of mEPSCs in hippocampal CA1 pyramidal neurons, and leads to a decrease in evoked synaptic potentials, LTP and spine maturity in CA1 basal dendrites, and to ultrastructural alterations in the post-synaptic compartment. These observations suggest that despite the expression of other TARP members in the hippocampus, including γ3 and high enrichment in γ8 [4], stargazin is required for normal spine development and for maintaining a full complement of functional synapses, specifically in CA1 basal dendrites. Our results are in line with experiments using *stargazer*/*γ8*-knock-out mice, which showed that AMPAR-mediated transmission in CA1 pyramidal neurons is further reduced, compared to the reduction observed in *γ8*-knock-out mice [39], despite the fact that CA1 pyramidal neurons from *stargazer* mice did not show alterations in the ratio of AMPA to NMDA EPSC amplitudes [40]. The synergistic reduction in AMPAR-mediated transmission in the *stargazer/γ8* double knock-out mice implies some degree of functional redundancy for the two TARPs. If mutated stargazin is expressed, its incorporation in AMPAR complexes, even if less efficient than WT stargazin, will thus exert pathogenic effects, as suggested by the reduction in the frequency of mEPSCs and in evoked potentials and spine maturation in basal CA1 dendrites that we observed in stargazin V143L mice. These results are also in agreement with electron microscopy data showing that at Schaffer collateral/commissural synapses in the CA1 hippocampal region the presence of stargazin correlates with higher density of AMPAR expression [41] and thus presumably with the presence of a higher number of functional synapses, and with a recent study showing a high enrichment of stargazin in hippocampal spines [42]. Our data show a specific effect of the stargazin V143L variant in spine maturation in basal dendrites in CA1 neurons, which was not observed in apical dendrites. Spine morphology changes may be secondary to alterations in AMPAR content. Indeed, we detected a decrease in the fEPSPs slope and in LTP in CA1 basal synapses. An alternative is that the stargazin V143L mutation impacts specific interactions that play a role in spine maintenance/maturation, and thus directly impacts spine morphology through changes in the actin cytoskeleton. One example of such a stargazin interactor is Arc/Arg3.1 [43], which regulates spine morphology and structural plasticity through regulation of actin dynamics (reviewed in [44]). Our data at this point do not provide a basis to distinguish between the two possibilities.

Significant change in the frequency, but not in the amplitude, of mEPSCs was detected in CA1 neurons in stargazin V143L mice, which is apparently at odds with previous work showing that stargazin V143L overexpression in cultured cortical neurons leads to decreased frequency and amplitude of mEPSCs [16]. However, one major advantage of analyzing synaptic currents in stragazin knock-in animals is that endogenous levels of expression of mutant stargazin are kept, thus avoiding the over-representation of mutated stargazin in association to AMPAR complexes, and preserving the subcellular distribution of the protein. Curiously, the decrease in the frequency of mEPSCs in CA1 neurons in the stargazin V143L knock-in model was accompanied by a significant decrease in fEPSPs slope and in LTP in CA1 basal synapses but not in CA1 apical synapses. These observations indicate that stargazin has a specific role in maintaining spine structure and synaptic function and plasticity in CA1 basal dendrites, which agrees with the higher expression levels of stargazin in the hippocampal *stratum oriens*, where basal dendrites are located, compared with the *stratum radiatum*. Altogether, our data suggest that, besides the well-described brain region- and cell type-specific roles of TARPs, there may be subfield-specific roles that are determined by the subcellular distribution pattern of different TARPs.

The V143L variant was found to be dephosphoryated in cortical PSDs isolated from homozygous stargazin V143L KI mice, compared to WT PSDs. Phosphorylation of stargazin in its C-terminal region disrupts electrostatic interaction between the membrane and stargazin C-tail [37], promotes the extension of the C-tail into the cytoplasm and binding to PSD95 [38], and triggers diffusional trapping of AMPARs at synaptic sites [31]. Stargazin phosphorylation has been proposed to regulate Hebbian forms of synaptic plasticity [12] and to mediate experience-dependent plasticity and synaptic scaling [10, 11]. The lower level of phosphorylation of stargazin-V143L compared to the WT protein likely underlies its higher membrane diffusion rate at the membrane and its impaired capacity in supporting AMPAR synaptic traffic. The low phosphorylation of stargazin V143L may also determine the sequestration of its C-terminal tail in the plasma membrane and thus impair it from undergoing liquid-liquid phase separation with PSD scaffold proteins [9]. Changes in hippocampal spine maturation and in the ultrastructure of hippocampal PSDs may thus be a consequence of defective stargazin V143L phosphorylation and may be reflected in the decreased number of functional synapses detected in our mEPSC analyses in CA1 hippocampal neurons. While it is likely that the aberrant stargazin V143L phosphorylation contributes to the physiological effects observed, our MD analysis, which does not consider post-translational modifications in stargazin, also suggests compromised function for the V143L stargazin variant.

In this study we found that the V143L mutation in stargazin KI^VL/VL^ mice leads to altered spatial memory and associative memory. These alterations in hippocampal-dependent cognitive behavior are likely to be related to the changes in mEPSC frequency, synaptic connectivity and plasticity, in spine maturity and in PSD ultrastructure that we identified in the hippocampus of these mice. We did not detect changes in social interaction in the three chamber test in stargazin V143L mice, but stargazin KI^VL/VL^ mice showed impairment in preference for social novelty, suggestive of either a perturbation in social memory or a lack of motivation for social novelty. Stargazin KI^VL/VL^ mice also displayed impaired motor learning in the rotarod, pointing to possible functional and structural alterations in the cerebellum. Given the elevated expression of stargazin in the cerebellum [4] and its non-redundant functions in cerebellar excitatory synapses in several cerebellum circuits [2, 7, 40], future studies should examine cerebellum circuit-specific dysfunction triggered by the ID-associated stargazin mutation. The cognitive and social behavioral dysfunctions displayed by stargazin V143L mice most likely arise from alterations in a combination of brain circuits, depending on the stargazin expression pattern and its synaptic roles in different cell types. Together, our data provide the first evidence for the causal implication of stargazin in the pathogenesis of neurodevelopmental disorders.

## Methods

### Molecular dynamics simulations

The three-dimensional (3D) structure of stargazin was constructed by homology modeling using the MODELLER package [45], the target sequence retrieved from UniProt [46] (Q9Y698) and template used from the GluA2:stargazin complex (PDB-ID: 6DLZ [26]). Molecular Dynamics (MD) simulations of AMPAR:stargazin WT and mutated form (V143L variant) were performed using GROMACS 2018.4 [47] and the CHARMM36 force field [48]. Systems were built using CHARMM-GUI [49, 50] membrane builder with a bilayer membrane of POPC:Cholesterol (9:1 ratio). Root mean square deviations (RMSD) and solvent-accessible surface area (SASA) calculations were performed using the Cα atoms by GROMACS package [45]. The cross-correlation analysis (CCA) was calculated by Bio3D R package [51] for residue-level dynamic analysis using the Cα trajectory. Free-energy calculations were performed using AMBER’s MMPBSA.py [52] as implemented in gmx_MMPBSA package [53].

### Primary cortical neurons, neuronal transfection and imaging

Primary cultures of rat cortical neurons were prepared from the cortices of E17 Wistar rat embryos, as previously described [54]. Neurons were transfected using a calcium phosphate-mediated transfection protocol [55]. Immunocytochemistry, quantum dots labeling, imaging and analysis were performed blind, and as described [56].

### Animal generation and maintenance

Stargazin V143L KI mice were generated by inserting a single nucleotide mutation in the third exon of the *Cacng*2 gene. The targeting vector was introduced through homologous recombination in R1 cells, as described previously [57]. The imaging, biochemical and behavioral analyses were performed in mice with 8–10 weeks and electrophysiology recordings were performed in 15–20 days-old animals. Both male and female animals were used; in the case where different conclusions were drawn for male and female animals (open field activity), results were plotted separately. All procedures and quantifications were performed by experimentalists blinded to animal genotype. Sample size estimates were based on previous literature. No randomization was applied. Procedures involving animals were performed according to the EU Directive 2010/63/EU guidelines and the experiments were approved by the institutional animal welfare body (ORBEA) and the national competent authority (DGAV).

### Behavior analyses

The object displacement test was performed in a 40 × 40 cm open field arena. In the first trial the animals acclimatized to the empty arena for 6 min. In the three following trials, the animals were allowed to explore, for 6 min, two different objects located in a fixed position. In the fifth trial, conducted 24 h later, one of the objects was displaced and the time spent exploring the non-displaced and the displaced object was evaluated. All other behavior tests were performed as described in [58].

### Electrophysiology

300 µm acute hippocampal sagittal slices were prepared from WT and stargazin V143L KI mice littermates, as previously described [58, 59]. Whole-cell voltage-clamp recordings were performed at a holding potential of −80 mV using a Multiclamp 700B amplifier, digitized at 20 kHz with Digidata 1550 A (Molecular Devices Corporation, USA), and acquired using Clampfit 10.7 software (Axon Instruments, USA) [58]. Data were analysed using Clampfit software (Axon Instruments, USA) using a template search method to detect events [60]. fEPSPs were evoked by stimulating axons in CA1 *stratum oriens* or in CA1 *stratum radiatum* at 0.05 Hz using a bipolar electrode and recorded in the same layer. An input-output curve was performed and the stimulation intensity was set to elicit 40–50% of the maximal response. LTP was induced by theta-burst stimulation (TBS; 10 bursts of 4 stimuli at 100 Hz with a burst frequency of 5 Hz) [61]. Fiber volley amplitude and synaptic response slopes were analyzed using Clampfit software. All electrophysiology experiments and analyses were done blind to the genotype.

### Labeling, detection and morphological classification of dendritic spines

To achieve sparse labeling of neurons in the hippocampus, we performed tail-vein injections in 4-week-old animals, with 5 µL of AAV9.Syn.eGFP.WPRE.bGH at a titer of 8.88 × 10^12^ (Penn Vector Core, University of Pennsylvania, USA). Four weeks post-injection, animals were sacrificed and the brains were collected and processed for neuronal imaging as described [58].

### Electron microscopy

Sample preparation and post-synaptic density parameter measurements were performed as previously described in [58].

### Biochemistry

Mice were anesthetized with isoflurane and euthanized by decapitation. Tissue lysates and post-synaptic density (PSD) isolations were carried out at 4 °C. Cortical lysates were subjected to the PSD isolation protocol previously described in [59]. Immunoprecipitation (IP) of stargazin was performed as previously described [62]. Lambda phosphatase (λ-PP) treatment of cortical PSD samples was performed using the λ-PP treatment kit from New England Biolabs (USA), according to the manufacturer’s instructions.

### Statistical analysis

The normality of population distributions was calculated for each experiment by comparison with a theoretical normal distribution using the Shapiro-Wilk normality test. According to this evaluation parametric or non-parametric tests were used, as described in the figure legends. For all tests, *p* < 0.05 was considered statistically significant. Outliers were identified and removed from the biochemical and behavioral analyses using the Grubbs test. Variance analysis following one-way ANOVA was performed using the Brown-Forsythe test. Analyses were performed using GraphPad 9.0 (Prism, USA). Details concerning number of independent experiments, statistical tests used, and *p-*values can be found in Table S1.

Additional details on the Materials and Methods are available in SI appendix.

## Supplementary information

Supplemental Material
