# Supplementary material for: Aberrant hippocampal transmission and behavior in mice with a stargazin mutation linked to intellectual disability
Source: Mol Psychiatry. 2022 Mar 7;27(5):2457–69. doi: 10.1038/s41380-022-01487-w (PMC9135633; doi:10.1038/s41380-022-01487-w)
Supplement: Supplementary file 1 — Supplemental Material [file 41380_2022_1487_MOESM1_ESM.docx]

**SUPPLEMENTAL INFORMATION**

**Aberrant hippocampal transmission and behavior in mice with a stargazin mutation linked to intellectual disability**

Caldeira GL*, Inácio AS*, Beltrão N, Barreto CAV, Rodrigues MV, Rondão T, Macedo R, Gouveia RP, Edfawy M, Guedes J, Cruz B, Louros SR, Moreira IS, Peça J, Carvalho AL

**SUPPLEMENTARY METHODS**

### Modeling the three-dimensional protein structure

The three-dimensional (3D) structure of stargazin was constructed by homology modelling using the MODELLER package([1](#_ENREF_1)), the target sequence retrieved from UniProt([2](#_ENREF_2)) (Q9Y698) and template used from the GluA2:stargazin complex (PDB-ID: 6DLZ([3](#_ENREF_3)); electron microscopy with 3.9 Å resolution; Human Organism; 99.5% sequence similarity). The best one hundred models from MODELLER([1](#_ENREF_1)) were evaluated by DOPE score, z-score([4](#_ENREF_4), [5](#_ENREF_5)), LGscore and MaxSub([6](#_ENREF_6)). The final model loops were further optimized. Due to the lack of a well-defined secondary structure with subsequent high conformational heterogeneity, the C-terminal of this protein, located at the intracellular level, was removed from the final model. The V143L stargazin mutation was built using the mutagenesis tool of PyMOL, creating the ID model. The 3D structure of GluA2 (Ligand-binding domain – LBD - and transmembrane domain – TMD) was also constructed using the MODELLER package([1](#_ENREF_1))and the subunit of AMPAR as the template (PDB-ID: 6DLZ), with the sequence P42262 from UniProt. The final model was selected using the previous criteria and the complexes AMPAR:stargazin (WT and V143L variant) were obtained by the superimposition of the stargazin and AMPAR models with 6DLZ structure. The final model 3D structure is illustrated in Figure 1b.

### Molecular dynamics simulations

Molecular Dynamics (MD) simulations of AMPAR:stargazin WT and mutated form (V143L variant) were performed using GROMACS 2018.4([7](#_ENREF_7)) and the CHARMM36 force field([8](#_ENREF_8)). The complex orientation in the membrane was obtained through the oriented crystal of GluA2:stargazin complex (PDB-ID: 6DLZ). Systems were built using CHARMM-GUI([9](#_ENREF_9), [10](#_ENREF_10)) membrane builder with a bilayer membrane of POPC:Cholesterol (9:1 ratio) to replicate the physiological environment. Each complex was solvated by a TIP3 water box and 0.15 M of NaCl. The final WT and mutated systems were constituted by 518.000 and 521.000 atoms, respectively.

The systems were subjected to an initial minimization to remove bad contacts using the steepest descent algorithm. Subsequently, they were heated using the Berendsen-thermostat at 310 K in the NVT ensemble over 7 ns, followed by an NPT ensemble of 20 ns with a semi-isotropic pressure coupling algorithm([11](#_ENREF_11)), which is used to keep the pressure constant of one bar. Long-range electrostatic interactions were treated by the fast smooth Particle-Mesh Ewald method([12](#_ENREF_12)). All bonds, involving hydrogen atoms within protein and lipid molecules were constrained using the linear constraint solver (LINCS) algorithm([13](#_ENREF_13)). Additionally, a cut-off distance of 12 Å was attributed to Coulombic and van der Waals interactions. Three independent replicas were run for each system during 0.5 μs, of which the first 0.15 μs of equilibration were left out of the further analysis.

Root mean square deviations (RMSD) calculations were performed using the Cα atoms by GROMACS package([1](#_ENREF_1)). The cross-correlation analysis (CCA), which tracks the movements of two or more sets of time series data relative to one another, was calculated by Bio3D R package([14](#_ENREF_14)) for residue-level dynamic analysis using the Cα trajectory. CCA analysis provides atomistic detail about the dynamic nature of proteins, and in particular allows the differentiation between regions that exhibit correlated or anticorrelated motions with others, in the same or in the opposite direction, respectively([15](#_ENREF_15)). The solvent-accessible surface area (SASA) analysis for each residue was performed using GROMACS package([7](#_ENREF_7)). These analyses were performed for the bound and unbound systems, and ΔSASA by residue was calculated as SASA_AMPAR:STG_ (SASA_GluA2_ + SASA_STG_). ΔSASA values, summed by substructure, provide another quantitative measure of conformational change upon protein coupling([16](#_ENREF_16)).

Free-energy calculations were performed using AMBER’s MMPBSA.py([17](#_ENREF_17)) as implemented in gmx_MMPBSA package([18](#_ENREF_18)). Parameters for Poisson-Boltzmann calculation were used as default except for i) the internal dielectric constant that was set as 20; ii) the ratio between the longest dimension of the rectangular finite-difference grid and that of the solute that was set to 1.25; iii) the ionic strength was set as 0.15 M; iv) the membrane dielectric constant was defined as 7; v) the membrane thickness as 31 Å.

**Primary cortical neurons**

Primary cultures of rat cortical neurons were prepared from the cortices of E17 Wistar rat embryos. Briefly after dissociation, the cortices were incubated with trypsin (0.06%, 10 min, 37ºC; GIBCO Invitrogen, USA) in Ca^2+^- and Mg^2+^-free HBSS (5.36 mM KCl, 0.44 mM KH_2_PO_4_, 137 mM NaCl, 4.16 mM NaHCO_3_, 0.34 mM Na_2_HPO_4_.2H_2_O, 5 mM glucose, 1 mM sodium pyruvate, 10 mM HEPES and 0.001% phenol red), washed 6 times with HBSS and then mechanically dissociated. After counted, the cells were plated, at a low density (0.3x10^6^ cells per 60 mm culture dish), in neuronal plating medium (MEM supplemented with 10% horse serum, 0.6% glucose and 1 mM pyruvic acid) in five poly-D-lysine (0.1 mg/ml) coated coverslips (18 mm). The medium was replaced, after 2 h, by Neurobasal medium supplemented with SM1 (StemCell Technologies, Canada), 0.5 mM glutamine and 0.12 mg/ml gentamicin. Neurons grew facing a confluent feeder layer of astroglial cells but were kept apart from the glial cells by wax dots placed on the coverslips([19](#_ENREF_19)). The cultures were treated with 5 μM cytosine arabinoside, two days after plating, to prevent the overgrowth of glial cells and were maintained in an incubator with 5% CO_2_, at 37ºC. Conditioned medium was partially replaced by fresh, SM1 supplemented neurobasal medium every 3 days. Primary cortical cultures were used for imaging.

**Transfection of cortical neurons**

Neurons were transfected using a calcium phosphate-mediated transfection protocol([20](#_ENREF_20)). A CaCl_2_ solution (2.5 M in 10 mM HEPES) was added, dropwise, to the diluted DNA. This solution was then added to the equivalent volume of HEPES-buffered transfection solution (274 mM NaCl, 10 mM KCl, 1.4 mM Na_2_HPO_4_, 11mM dextrose and 42 mM HEPES, pH 7.2). The DNA precipitates were added, dropwise, to the coverslips in conditioned medium and 2 mM of kynurenic acid. The cultures were incubated for 2 h at 37ºC and 5% CO_2_. The DNA precipitates were dissociated by incubating the cells with acidified medium, for 15 min at 37ºC and 5% CO_2_. Coverslips were then transferred to the original astroglial-containing dish.

**Immunocytochemistry and imaging**

In order to stain surface proteins, live cells were incubated with anti-GluA (MAB2263; Millipore, USA) primary antibody diluted in conditioned medium for 10 min and fixed for 15 min in 4% sucrose/ 4% paraformaldehyde in PBS at room temperature. Following 3 washes with PBS, the cells were incubated with the secondary antibody (Molecular Probes, USA) diluted in 3% BSA, in PBS, for 45 min, 37ºC. After 6 washes with PBS, cells were permeabilized for 5 min with 0,25% Triton X-100, in PBS at 4ºC. Unspecific staining was blocked by incubation with 10% (w/v) BSA in PBS for 30 min, at 37ºC. In order to label PSD95 (MA1-045; Thermo Scientific, USA) and MAP2 (ab5392; Abcam, UK), neurons were incubated with the primary antibodies diluted in 3% BSA in PBS for 2 h at 37ºC or overnight at 4ºC. Before and after incubating with the secondary antibodies, also diluted in 3% BSA in PBS, for 45 min, 37ºC, cells were washed 6 times with PBS. Coverslips were mounted in DAKO fluorescent mounting medium. The imaging was performed using a Zeiss Axiovert 200 M microscope and a 63X (NA1.4) oil objective. Blind-to-condition quantification was performed in ImageJ (NIH, USA) analysis software, with a macro that automatized quantification steps. The region of interest (ROI) was chosen randomly, by using MAP2 and/or GFP staining to confirm that the selected dendrite was from a transfected neuron. The threshold was defined to include detectable clusters and the signal intensity of the particles of the selected area was analyzed. Synaptic puncta were defined by their colocalization with PSD95.

**Quantum dots labeling, imaging and analysis**

Low-density 12 days *in vitro* (DIV) cells were co-transfected with plasmids encoding Homer-GFP, for synapse identification, and HA-tagged WT stargazin or the V143L stargazin variant. At DIV 14, cells were incubated for 10 min at 37ºC with anti-HA antibody (3F10; Roche, Switzerland) (1:3000) diluted in conditioned medium. After one washing step, anti-rat IgG conjugated QD655 (diluted 1:10 in PBS) was diluted in conditioned medium with 2% BSA (1/2000) and added to cells for 5 min at 37°C. All washes were performed in ECS containing 145 mM NaCl, 5 mM KCl, 10 mM Glucose, 10 mM Hepes, 2 mM CaCl_2_ and 2 mM MgCl_2_, supplemented with 2% BSA at 37°C. Neurons were mounted in an open chamber (K.F. Technology SRL, Italy) and imaged in ECS. Single-particle tracking was performed as in([21](#_ENREF_21)). Cells were imaged at 37°C on an inverted microscope (Axio Observer Z1, Carl Zeiss, Germany) equipped with a Plan Apochromat 63X (NA 1.4) oil objective. Homer1C-GFP signal was detected by using an HXP fluorescence lamp (For QDs: excitation filter 425/50 and emission filters 655/30, Chroma). Fluorescent images from QDs were obtained with an integration time of 50 ms with up to 600 consecutive frames. Signals were recorded with a digital CMOS camera (ORCA Flash 4.0, Hamamatsu, Japan). The tracking of single QDs was performed using the Metamorph and Matlab (Mathworks Inc., Natick, USA) software tools. Due to random blinking, the trajectories were not continuously tracked, instead, when the positions before and after the dark period were compatible with borders set for maximal position changes between consecutive frames and blinking rates, the subtrajectories of the same molecule were reconnected. MSD curves were calculated for reconnected trajectories of at least 20 frames. The QDs were considered synaptic if colocalized with Homer-1c dendritic clusters for at least five frames. Diffusion coefficients were calculated by a linear fit of the first 4–8 points of the mean square displacement (MSD) plots versus time depending on the length of the trajectory within a certain compartment. The resolution limit for diffusion was 0.0075 μm^2^/s as determined by([22](#_ENREF_22)), whereas the resolution precision was ~40 nm.

**Animal generation and maintenance**

Stargazin V143L KI mice were generated by inserting a single nucleotide mutation in the third exon of the *Cacng*2 gene. The targeting vector was introduced through homologous recombination in R1 cells, as described previously([23](#_ENREF_23)). Mice were viable and born at the expected Mendelian ratio. Genotyping was performed by PCR from mouse ear or tail DNA using a forward primer for the WT allele (AAGGGACCCTCCGTCCTCTC), a forward primer for the KI allele (GGGCCCGGTGCAATACACGC) and a reverse primer for both the reactions (CATCGGGCATGGATCCTCAGTTC). Mice were maintained at 22ºC and 60% humidity under a 12 h light/dark cycle. Food and water *ad libitum* were provided. The imaging, biochemical and behavioral analyses were performed in mice with 8-10 weeks and electrophysiology recordings were performed in 15-20 days-old animals. In all experiments except spine analysis (males only) and electron microscopy (females only), both male and female animals were analyzed. Data plotting shows female and male animals with different representations. In the case where different conclusions were drawn for male and female animals (open field activity), results were plotted separately.

All behavioral tests and quantifications were performed by experimentalists blinded to animal genotype. Sample size estimates were based on previous literature. No randomization was applied. All the procedures involving animals were performed according to the guidelines established by the European Union Directive 2010/63/EU and the experiments were previously approved by the institutional animal welfare body (ORBEA) and the national competent authority (DGAV).

**Nissl Staining**

Eight-week-old mice were anesthetized with isoflurane and perfused with ice cold PBS followed by 4% paraformaldehyde in PBS. Whole brains were kept in 4% paraformaldehyde in PBS overnight and then transferred to a 30% sucrose in PBS solution for at least 24 h. Brains were sliced in the cryostat (Thermo Cryostar NX50, Thermo Fisher Scientific, USA) to obtain 50 μm coronal slices which were mounted in gelatin-coated slides. The slides were briefly washed with water and then submersed in a cresyl violet solution, for 5 min. After 2 washes with water, the slices were decolored with 100% ethanol for 2 min and incubated for 2 min in xylene before mounting with Permount mounting medium (Fisher Scientific, USA). Brain slices were digitized using a Zeiss Axio Scan.Z1 slide scanner (Carl Zeiss, Germany) equipped with a Plan Apochromat 20X (NA 0.8) air objective.

**Immunohistochemistry**

For stargazin immunofluorescence staining, 50 μm coronal and sagittal brain slices were prepared as described above. Free-floating sections were rinsed 3 times in PBS for 10 min and then permeabilized and blocked for 1 h at room temperature with 0.25% Triton X-100 and 5% goat serum in PBS. After that, slices were incubated overnight at room temperature with the primary antibody (AB_2571844, Frontier Institute Co., Japan) diluted 1:200 in 0.25% Triton X-100 and 2% goat serum in PBS, followed by 3 washes with 0.25% Triton X-100 in PBS. Sections were incubated with the secondary antibody (anti-rabbit Alexa Fluor 568, Molecular Probes, USA) diluted 1:500 in 0.25% Triton X-100 and 2% goat serum in PBS, at room temperature for 2 h. Nuclei were visualized by staining with 1μg/mL Hoechst 33342 in PBS for 5 min at room temperature. Lastly, after 3 washes of 10 min in PBS, the sections were mounted in gelatinized slides using Dako mounting medium (Glostrup, Denmark). Images were acquired on a Carl Zeiss Axio Imager Z2 upright widefield microscope (Carl Zeiss, Germany) using a Plan-Apochromat 20X (NA 0.8) air objective or in an LSM 710 Confocal microscope (Zeiss, Germany) with a Plan Apochromat 63X (NA 1.4) oil objective.

##### RNA extraction and qPCR

Total RNA was extracted from 8 weeks-old mice cortices and hippocampi with miRCURY™ RNA Isolation Kits - Cell & Plant (Exiqon, Denmark), according to manufacturer’s instructions. RNA concentration was determined using a NanoDrop 2000c/2000 UV-Vis Spectrophotomer (Thermo Fisher Scientific, USA). cDNA was synthesized by reverse transcription NZY First-Strand cDNA Synthesis Kit (NZYtech, Portugal), according to manufacturer instructions, in T100 thermal cycler (Bio-Rad, USA).

Primers for real-time PCR were designed using the Universal Probe library (Roche Life Science, Switzerland). Gene expression analysis was performed using SsoFast SuperMix (Bio-Rad; USA), according to manufacturer’s specifications. The fluorescence was measured using the iQ5 Multicolor Real-Time PCR Detection System (BioRad, USA). Data was analyzed with the GenEx software (MultiD Analyses, Sweden). The constitutively expressed housekeeping gene encoding HPRT was used as control.

**Behavior analyses**

***Contextual fear conditioning test***

The contextual fear conditioning test was performed in an electrified wire-bottom 20x20 cm cage. On the first day, the animal was placed for 2 min in the cage before receiving a 2s-long foot shock of 0.5 mA. After 30 s, a second foot shock with the same magnitude and duration was administered, another 30 s later the animal was removed from the cage. After 24 h, the animal was placed in the same cage and it was recorded for 3 min. The time spent in freezing behavior was scored using the Observer XT 12 software (Noldus, Netherlands).

***Rotarod test***

Motor function and learning was evaluated using the accelerated rotarod (Med Associates, USA), 4 to 40 rpm in 5 min. The time withstood in the rotating beam was averaged for three successive trials in each day and evaluated for 2 days. An improvement in the performance in the second day was considered motor learning.

***The three-chamber test***

The three-chamber test was evaluated in a tripartite arena (Stoelting, USA). The test was split into three trial epochs. In the first trial part, animals were allowed to freely explore the three chambers of the arena for 20 min. In the second trial part animals were allowed to voluntarily interact with an empty gridded cage recipient or with a similar cage recipient containing a stranger animal, for 20 min. The preference index for social behavior (S1:E) was determined as follows: $\frac{Sniffing time\left( Stranger 1 \right) - Sniffing time\left( Empty cage \right)}{Sniffing time\left( Stranger 1 \right) + Sniffing time\left( Empty cage \right)}\times100$. In the third part of the trial, which also lasted 10 min, a second stranger was placed in the previously empty cage recipient. The preference index for social novelty (S2:S1) was determined as follows: $\frac{Sniffing time\left( Stranger 2 \right)- Sniffing time(Stranger 1)}{Sniffing time(Stranger 2)+ Sniffing time(Stranger 1)}\times100$. The time spent in close proximity sniffing with the gridded recipients was evaluated for the first half of the second and third trials using the Observer XT 12 software (Noldus, The Netherlands).

***Open field test***

In the open field test animals were allowed to freely move, for 1 h, in an opaque arena (40x40 cm). The room was illuminated by white LED lamps at 100 lux. The locomotor activity (total distance traveled, instant velocity and time in center zone) was quantified with the Bonsai software using the recorded videos.

***Elevated-plus maze test***

The EPM consists of a cross-shaped platform, containing four arms, which is elevated from the ground; two of the arms are enclosed by walls whereas the other two are open and thus subjected to bright illumination (100 lux). The animals initiated the test in the center of the maze and their movement was recorded for 10 min. The preference for the open or closed arms, as well as the time spent in the center was evaluated using the Bonsai software.

***Forced-swimming test***

FST was conducted by placing the mice in 2 L glass beakers filled with 1.6 L of water at 24 ± 1°C. The total duration of immobility over the 6 min observation period was scored using the Observer XT 12 software (Noldus, Netherlands). Immobility was defined as the lack of motion of the whole body, except for small movements necessary to keep the animal’s head above the water.

***T-maze spontaneous alternation test***

The spontaneous alternation test was performed in a T-shaped maze (30x10 cm), illuminated with 15-20 lux. The mice were introduced in the start arm and allowed to choose one of the other arms. After the animals entered one of the arms, it was enclosed in that area with a sliding door, for 30 s. The animal was then removed from the maze for 30 s more and re-placed in the start arm. If the animals chose the previously unexplored arm, spontaneous alternation was considered. A total of five trials were conducted in 2 consecutive days.

***Nest-building test***

Mice were transferred to individual standard home cages (20×26×13 cm) with corn bedding, 1 h before the dark phase. A cotton disk was added to the cage and, 16 h later, the nests were evaluated by blind-to-genotype observers, using a 5-point rating([24](#_ENREF_24)).

**Slice preparation and electrophysiological recordings**

WT and stargazin V143L KI mice littermates (P15-P20) were deeply anesthetized with isofluorane and transcardially perfused with ice-cold sucrose cutting solution (212.7 mM sucrose, 2.6 mM KCl, 1.23 mM NaH_2_PO_4_, 26 mM NaHCO_3_, 10 mM glucose, 3 mM MgCl_2_, 1 mM CaCl_2_, pH 7.4, 300–320 mOsm) oxygenized with carbogen (95% O_2_ and 5% CO_2_). The brain was quickly removed and immersed in oxygenated ice-cold sucrose cutting solution. 300 µm acute hippocampal sagittal slices were prepared using a vibratome (Leica VT1200s, Leica Microsystems, USA). The slices were collected and transferred to a submersion holding chamber with artificial cerebrospinal fluid (aCSF; 125.1 mM NaCl, 2.5 mM KCl, 1.1 mM NaH_2_PO_4_, 25 mM NaHCO_3_, 25.0 mM glucose, 2 mM MgSO_4_, 2 mM CaCl_2_, pH 7.4, 300–310 mOsm) continuously oxygenated with carbogen, at 32°C, for 30 min. After that, slices were allowed to further recover for 1 h at room temperature, in oxygenated aCSF, before recording.

CA1 pyramidal neurons were visualized under infrared-differential interference contrast (IR-DIC) microscopy using an upright microscope (Axio Examiner.D1, Zeiss, Germany). Whole-cell voltage-clamp recordings were performed at a holding potential of -80 mV using a Multiclamp 700B amplifier, digitized at 20 kHz with Digidata 1550A (Molecular Devices Corporation, USA), and acquired using Clampfit 10.7 software (Axon Instruments, USA). Slices were kept in a recording chamber perfused with oxygenated aCSF (2–3 mL/min), at 30°C, supplemented with 1 µM TTX, 100 µM picrotoxin and 50 µM D-APV, to isolate AMPAR-mediated mEPSC. Borosilicate glass recording pipettes (3-5 MΩ) were filled with a Cs-based solution (115.0 mM CsMeSO_3_, 20.0 mM CsCl, 2.5 mM MgCl_2_, 10.0 mM HEPES, 0.6 mM EGTA, 10 mM Na-phosphocreatine, 4 mM ATP sodium salt, 0.4 mM GTP sodium salt, pH 7.3, 295–300 mOsm). Data were filtered at 2 kHz. mEPSCs were recorded for 5-8 min. Cells were discarded if Ra was higher than 25 MΩ or if holding current or Ra changed more than 20%. Data were analysed using Clampfit software (Axon Instruments, USA) using a template search method to detect events([25](#_ENREF_25)). The template was generated by averaging approximately 30 events and the template match threshold was set to 4. The same number of events was analyzed for each cell.

For extracellular field recordings, the aCSF composition (mM) was the following: 130.9 NaCl, 2.5 KCl, 1.1 NaH_2_PO_4_, 24.0 NaHCO_3_, 12.5 glucose, 2 MgSO_4_, 2 CaCl_2_, pH 7.4, 300–310 mOsm. fEPSPs were evoked by stimulating axons in CA1 *stratum oriens* or in CA1 *stratum radiatum* at 0.05 Hz using a bipolar electrode (100 µs stimulus; Bowdoin, ME, USA) connected to a stimulator Digitimer model DS3 (Digitimer, UK) and recorded in the same layer. Recordings were performed at 25°C in a recording chamber constantly perfused with oxygenated aCSF (2–3 mL/min). The recording pipette was filled with aCSF (2-4 MΩ). An input-output curve, starting at 20 µA with 10 µA increments, was performed and the stimulation intensity was set to elicit 40-50% of the maximal response. Only slices displaying a stable signal response over a period of 10 min were used. Short-term synaptic plasticity was assessed by measuring paired-pulse facilitation (PPF) using a standard protocol, as previously described([26](#_ENREF_26)). LTP was induced by theta-burst stimulation (TBS; 10 bursts of 4 stimuli at 100 Hz with a burst frequency of 5 Hz)([27](#_ENREF_27)). A baseline was recorded in the current-clamp mode with a single stimulation at 0.05 Hz (100 µs stimulus) for 15 min immediately before TBS. Changes in fEPSPs were recorded at 0.05 Hz for 60 min after TBS. Recordings were filtered at 0.1 Hz-1 kHz and digitized at 10 kHz. For each data point three individual traces were averaged. Fiber volley amplitude and synaptic response slopes were analyzed using Clampfit software. All electrophysiology experiments and analyses were done blind to the genotype.

##### Labelling hippocampal neurons for morphological analysis

To achieve sparse labelling of neurons in the hippocampus, we performed tail-vein injections in 4-week-old animals, with 5 µL of AAV9.Syn.eGFP.WPRE.bGH at a titer of 8.88x10^12^ (Penn Vector Core, University of Pennsylvania, USA) diluted in sterile PBS to a final volume of 100 µL. Four weeks post-injection, animals were sacrificed and the brains were collected and processed for neuronal imaging as already described. Brains were sliced in the cryostat to obtain 100 μm serial coronal slices and the GPF fluorescence signal was enhanced by performing immunostaining against GFP. Sections were then mounted in gelatinized slides using Vectashield with DAPI (Vector Laboratories, USA).

***3D neuronal reconstruction and Sholl analysis***

CA1 pyramidal neurons expressing GFP were randomly selected and imaged in an LSM 710 Confocal microscope (Zeiss, Germany) using a Plan-Apochromat 20X (NA 0.8) air objective. Six neurons selected from at least four different sections were analyzed per animal. 3D neuronal reconstructions and morphometric analysis were performed using Imaris software (Bitplane, Switzerland). Sholl analysis was performed by quantifying the number of intersections between dendrites and concentric spheres with a radius increment of 10 μm from the cell soma. The total length of basal and apical dendrites was also determined. Image acquisition and analysis was performed by a blind-to-genotype observer.

***Detection and morphological classification of dendritic spines***

Images of secondary basal and apical dendrites from CA1 pyramidal neurons expressing GFP were acquired in an LSM 710 Confocal microscope (Carl Zeiss, Germany) with a Plan Apochromat 63X (NA 1.4) oil objective. The dendritic segments imaged were randomly selected from at least four different sections. Per each animal, eight basal and eight apical dendritic segments, of approximately 20 µm, from different cells were imaged. The z-stack images were deconvolved using Huygens software (Scientific Volume Imaging, Netherlands) and spines were visualized and identified using Imaris software (Bitplane, Switzerland). Spines were manually categorized into five groups based on its morphology: mushroom (defined neck and a large head), stubby (without a defined neck), branched (cup-shaped; with a head protrusion; with multiple heads), thin (thin neck and small head) and filopodia (without a defined head). Image acquisition and analysis was performed by a blind-to-genotype observer.

##### Electron microscopy

Sample preparation and post-synaptic density parameter measurements were performed as previously described in([26](#_ENREF_26)). Eight-week-old mice were anesthetized with isofluorane and transcardially perfused with ice-cold PBS followed by 4% paraformaldehyde. Cortices and hippocampi were dissected, and small punches of tissue were left overnight in PFA 4% and then transferred into a 2.5% glutaraldehyde solution in 0.1 M sodium cacodylate buffer (pH 7.2), where they were kept at 4°C overnight. The tissue was then rinsed in a cacodylate buffer and post-fixed with 1% osmium tetroxide for 1 h. After rinsing in buffer and distilled water, 1% aqueous uranyl-acetate was added to the tissues, in the dark, during 1 h for contrast enhancement. Following rinsing in distilled water, samples were dehydrated in a graded acetone series (70–100%) and then impregnated and included in Epoxy resin (Fluka Analytical, Switzerland). Ultrathin sections (70 nm) were mounted on copper grids and observations were carried out on a FEI-Tecnai G2 Spirit Bio Twin at 100kV. PSD measurements were performed using ImageJ (NIH, USA) by a blind-to-genotype observer.

##### Tissue lysates and post-synaptic density isolations

Eight-week-old WT, stargazin KI^+/VL^ and KI^VL/VL^ mice were anesthetized with isoflurane and euthanized by decapitation. Tissue lysates and post-synaptic density (PSD) isolations were carried out as described below. All procedures were performed at 4ºC.

###### *Hippocampal lysates*

Hippocampi from WT, KI^+/VL^ and KI^VL/VL^ mice were mechanically homogenized in TEEN buffer (25 mM Tris pH 7.4, 1 mM EDTA, 1 mM EGTA, 150 mM NaCl and 1% Triton X-100, supplemented with 1 mM DTT, 0.2 mM PMSF, 1 µg/ml CLAP (1 mg/ml of Chymostatin, Leupeptin, Antipain and Pepstatin), 5 mM NaF and 0.1mM Na_3_VO_4_), using a motor driven glass-Teflon homogenizer at 900 rpm (50 strokes). Hippocampal homogenates were centrifuged at 700 g for 10 min and the supernatants were collected and sonicated using an ultrasonic probe for 60 s (6 pulses of 5 s). The samples were again centrifuged at 21100 x g for 10 min and the supernatants were collected.

###### *Whole brain lysates and PSD isolations*

Cortices and whole brain samples from WT, KI^+/VL^ and KI^VL/VL^ were dissected and homogenized in HEPES A buffer (4 mM HEPES pH 7.4, 0.32 M sucrose, supplemented with 1 mM DTT, 0.2 mM PMSF, 1 µg/ml CLAP, 5 mM NaF and 0.1mM Na_3_VO_4_), using a motor driven glass-Teflon homogenizer at 900 rpm (50 strokes). These homogenates were centrifuged at 700 x g for 15 min. The whole brain lysates and a fraction of the cortical lysates were collected in 2% SDS and 2.5 M Urea and stored at -80ºC for later analysis. The remaining cortical lysate was subjected to the PSD isolation protocol previously described in([28](#_ENREF_28)). Briefly, the lysates were centrifuged at 18000 x g for 15 min, resulting in the crude synaptosomal pellet, which was re-homogenized in HEPES A buffer and further centrifuged at 25000 x g for 20 min. This yielded the lysed synaptosomal membrane pellet. A fraction of this synaptosomal membrane lysate (SML) was collected in HEPES B buffer (50 mM HEPES pH 7.4, 2 mM EDTA, 0.5% Triton X-100, supplemented with 1 mM DTT, 0.2 mM PMSF, 1 µg/ml CLAP, 5 mM NaF and 0.1 mM Na_3_VO_4_) and 2% SDS and stored at -80ºC for later analysis. The remaining synaptosomal membrane fraction was resuspended and incubated in HEPES B buffer for 15 min and centrifuged at 32000 x g for 20 min. The resulting pellet was resuspended and incubated in HEPES B buffer for 15min and centrifuged at 200000 x g for 20 min. The resulting pellet, consisting of the PSD fraction, was collected in HEPES B buffer and 2% SDS, 2.5 M urea and stored at -80ºC for later analysis.

***Cortical lysates for immunoprecipitation***

Cortical lysates for immunoprecipitation were obtained as previously described**(**[**29**](#_ENREF_29)**)**. Briefly, fresh cortices from WT or KI^VL/VL^ littermates were mechanically homogenized in 5 ml of buffer A (20 mM HEPES, 0.15 mM EDTA, 0.4 mM EGTA, 10 mM KCl, pH 7.5, supplemented with 1 mM DTT, 0.2 mM PMSF, 1 µg/ml CLAP, 5 mM NaF, 0.1 mM Na_3_VO_4_) followed by sonication and then centrifuged for 10 min at 860 × g. The resulting supernatant was centrifuged for 30 min at 17000 × g. Buffer A was supplemented with 15% sucrose and 5 ml was used to homogenize each pellet with 20 strokes, which was further centrifuged for 10 min at 860 × g to remove genomic DNA. The brain membranes present in the supernatant were centrifuged again for 30 min at 17000 × g. The pellets were solubilized in buffer B (20 mM HEPES, 1% Triton-X100, 150 mM NaCl, 0.15 mM EDTA, 4 mM EGTA, pH 7.5, with the same cocktail of protease and phosphatase inhibitors) with 20 dunces with a potter and centrifuged at 17000 x g for 45 min, yielding the cortical lysates.

##### Stargazin immunoprecipitation

Immunoprecipitation (IP) of stargazin was performed as previously described([29](#_ENREF_29)). Briefly, protein concentration of cortical lysates was quantified with a Bicinchoninic Acid (BCA) assay (Fisher Scientific, USA). The lysates (500 µg) were incubated with the anti-stargazin antibody (IP+; AB-9876,2 µg, Merck Millipore, USA) or with normal Rabbit polyclonal IgG (IP-; 12-370,2 µg, Merck Millipore, USA) for 1 h at 4°C under rotation and then incubated overnight with 50 µl of protein-A Sepharose at 4°C. Resin was washed with 1 ml of buffer B and 0,5 ml of the same buffer supplemented with 500 mM NaCl. Beads were resuspended in 50 µl of 2X denaturing buffer (62.5 mM Tris·HCl pH 6.8, 10% Glycerol, 2% SDS, 0.01% bromophenol blue, and 5% β-mercaptoethanol).

##### Lambda phosphatase treatment

Lambda phosphatase (λ-PP) treatment of cortical PSD samples was performed using the λ-PP treatment kit from New England Biolabs (USA), according to the manufacturer’s instructions. In brief, cortical lysates from WT, stargazin KI^+/VL^ and KI^VL/VL^ mice were obtained according to the previously described protocol, without the supplementation with the phosphatase inhibitors NaF and Na_3_VO_4_. The lysates were then divided into two groups: treated and untreated samples. The cortical lysates from the untreated group were supplemented with 5 mM NaF and 0.1mM Na_3_VO_4_. Samples from the treated group were supplemented with 12.5% of NEBuffer for Protein MetalloPhosphatases (PMP), 12.5% of 10 mM MnCl_2_ and 2.5% of Lambda Protein Phosphatase. All samples from both groups were then incubated at 30°C for 30 min and processed according to the previously described PSD isolation protocol. This experiment was run twice using samples from independent animals.

##### SDS-PAGE and Western blot

Protein quantification was performed using the BCA assay (Fisher Scientific, USA). Samples were denatured with sample buffer 5X (NZYTech, Portugal) and resolved by SDS-PAGE in Tris-glycine-SDS buffer (25 mM Tris, 192 mM glycine, 0.1 % SDS, pH 8.3) in an 11% polyacrylamide gel. Stargazin immunoprecipitation samples were resolved in 4-20% Mini-PROTEAN® TGX™ Precast Protein Gels (BioRad, USA) in the same Tris-glycine-SDS buffer. All SDS-PAGE gels were subjected to an overnight electrotransfer (40 V, 4°C) to a PVDF membrane (Merck Millipore, USA). The membranes were then blocked using a 5% milk solution in TBS (20 mM Tris, 137 mM NaCl, pH 7.6) supplemented with 0.1% Tween-20 (TBS-T) for 1 h at room temperature (RT). After blocking, the membranes were incubated with the primary antibodies against stargazin (AB-9876, Merck Millipore; 1:750 in 3% Milk TBS-T), GluA1 (MAB2263, Merck Millipore, USA; 1:1000 in 5% Milk TBS-T), GluA2 (MAB397, Merck Millipore, USA; 1:1000 in 5% Milk TBS-T) and PSD95 (MA1-045, Thermo Fisher Scientific, USA; 1:1000 in 5% milk TBS-T) for 2 h at RT. The membranes were washed 3 times for 10 min in TBS-T and then incubated with the appropriate alkaline phosphatase-conjugated secondary antibody (#115-055-146 or #211-055-109, Jackson ImmunoResearch, USA; 1:10000 in 5% milk TBS-T) for 45 min at RT. Following 3 washes in TBS-T, membranes were developed with the alkaline phosphatase substrate ECF (GE Healthcare, USA) and the fluorescent signal was acquired using a ChemiDoc Gel Imaging System (BioRad, USA). The results were analyzed using ImageJ (NIH, USA).

**SUPPLEMENTARY FIGURES**


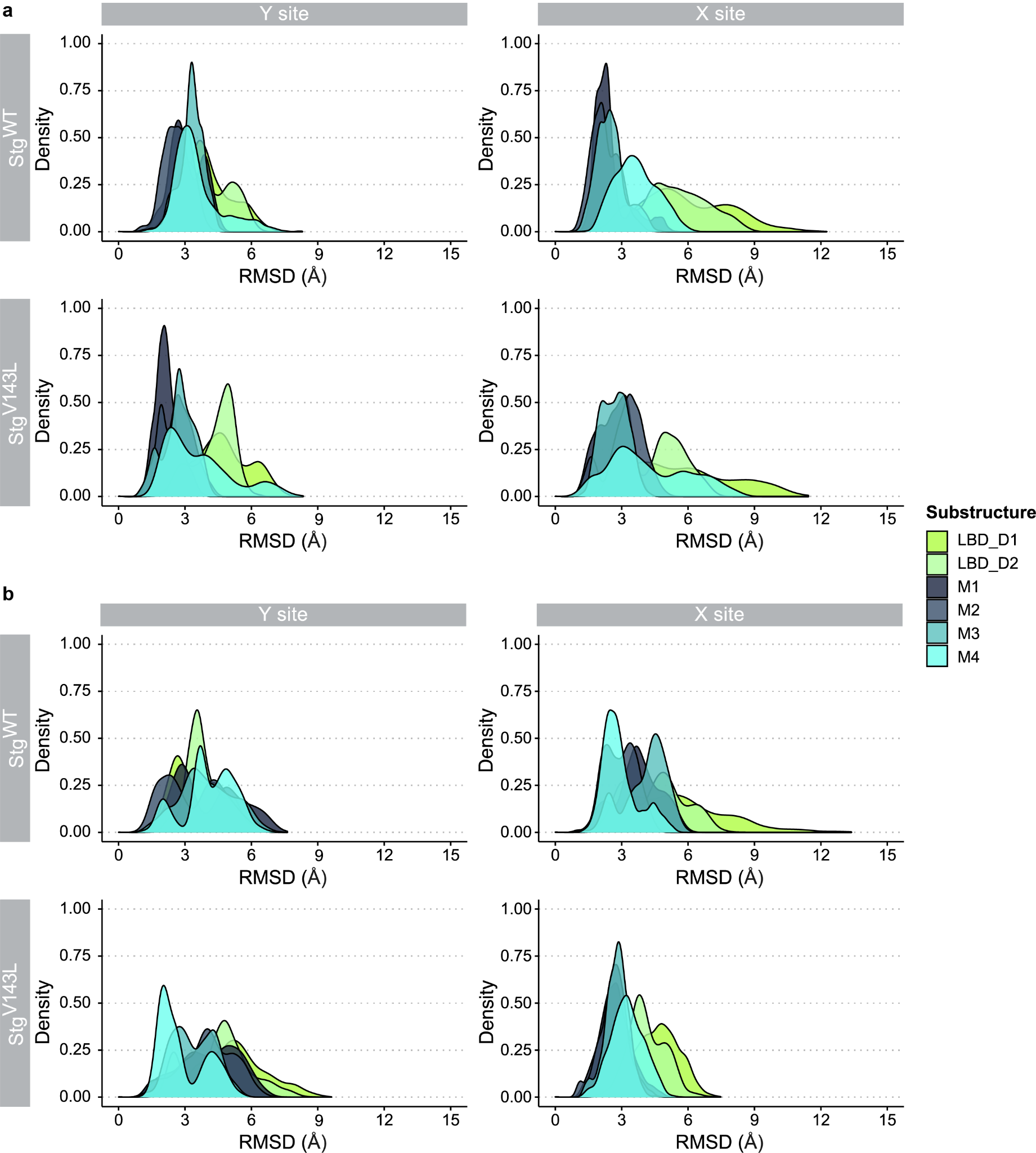


**Figure S1. RMSD density plots for Cα atoms of *Main GluA2* and *Auxiliary GluA2*.**

(**a**) RMSD values were calculated for every substructure of *Main GluA2* and (**b**) Secondary *GluA2* for MD simulations of both stargazin WT- and stargazin V143L-containing systems. Colors from Figure 1 were used for substructures of GluA2. At both X and Y sites, the M1 and M2 show higher deviation and conformational flexibility in the mutated systems, while M3 and M4 show lower deviation and flexibility, particularly for the mutated X site.

**
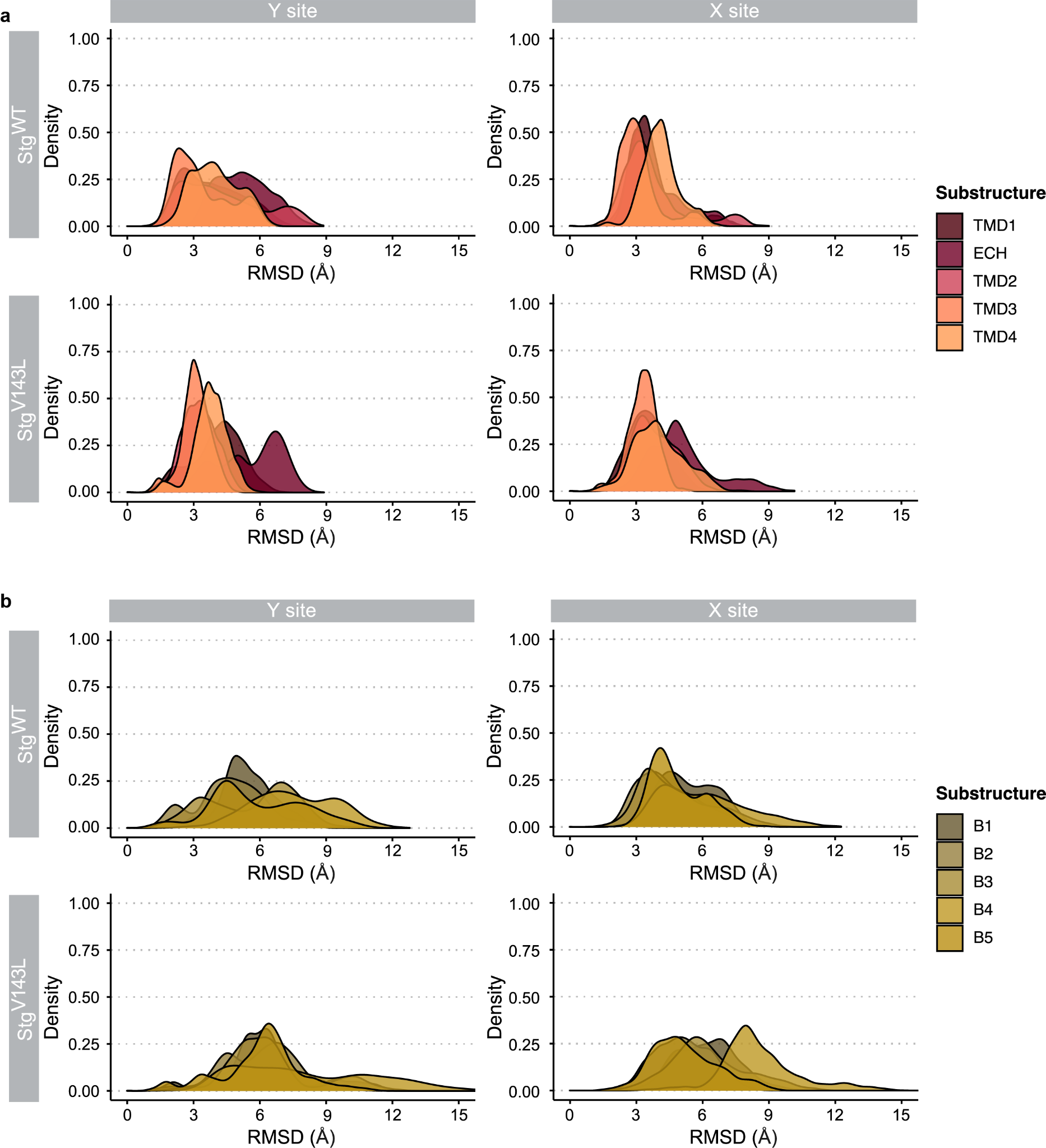
**

**Figure S2. RMSD density plots for Cα atoms of stargazin helices and stargazin β-strands.**

(**a**) RMSD values were calculated for every helix and (**b**) β-strand of stargazin for MD simulations of both stargazin WT and stargazin V143L-containing systems. Colors from Figure 1 were used for substructures of stargazin. TMD3 and TMD4 show higher density for lower RMSD values, and therefore lower conformational flexibility in the mutated system. In the case of the β-strands, their RMSD values were higher for the mutated system at both sites, which shows a higher conformational flexibility of this system, especially for β4 and β5.

**
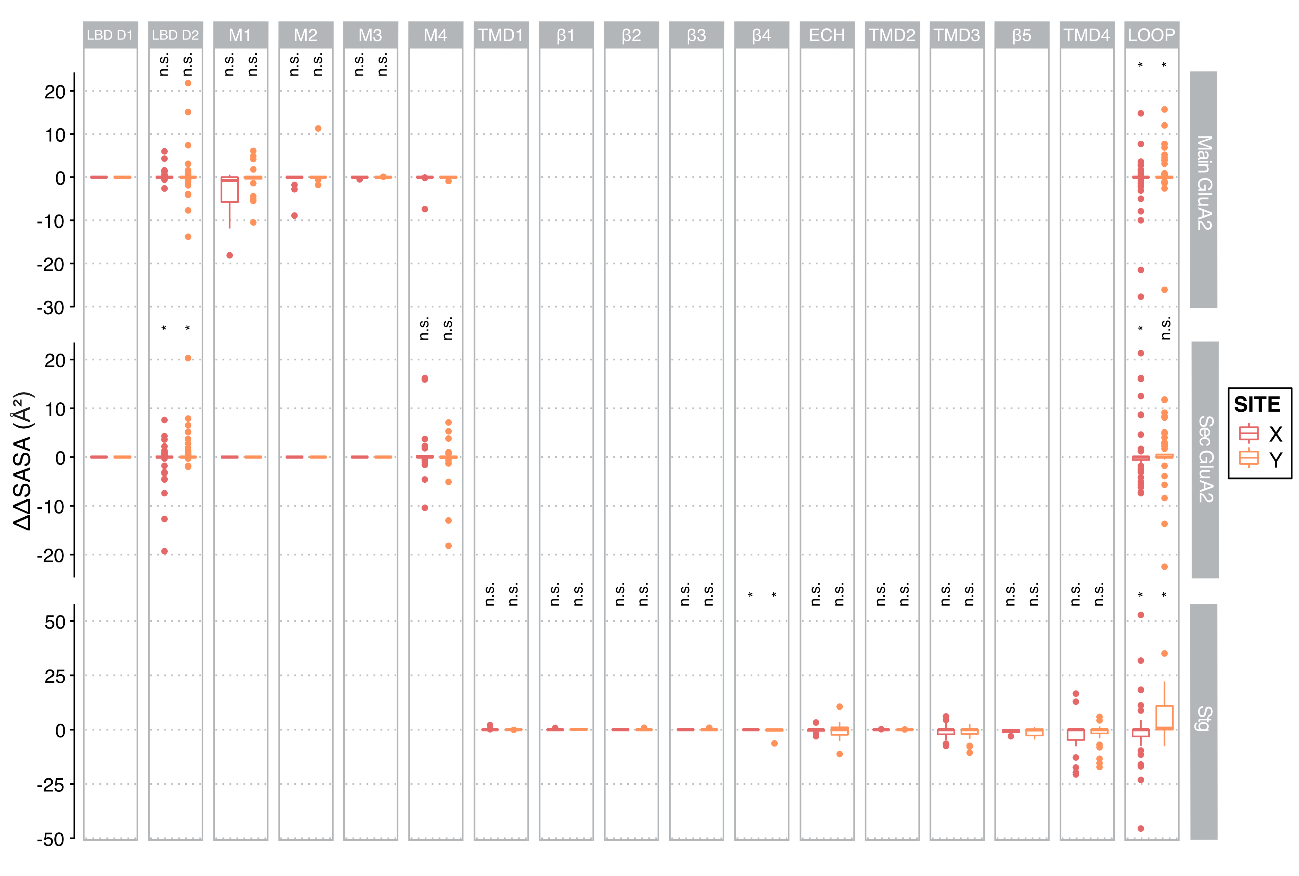
Figure S3.** ΔΔ**SASA values for AMPAR:stargazin complex per substructure.**

Mean ΔΔSASA of each residue was calculated as ΔΔSASA = ΔSASA_V143L_ – ΔSASA_WT_. Residues were grouped by substructure. Wilcoxon signed- rank test was used to compare ΔΔSASA values to zero (represents no change between V143Land WT), **p*<0.05. X site – red; Y site – orange. For both sites and systems, M1 in the *Main GluA* and M4 in the *Secondary GluA* display the lowest ΔSASA values (highest contribution) in the TM region, with M1 presenting a different behavior between the two sites (data not shown). In stargazin, the substructures with the highest contribution for the interface were TMD3 and TMD4 in the TM region (data not shown).


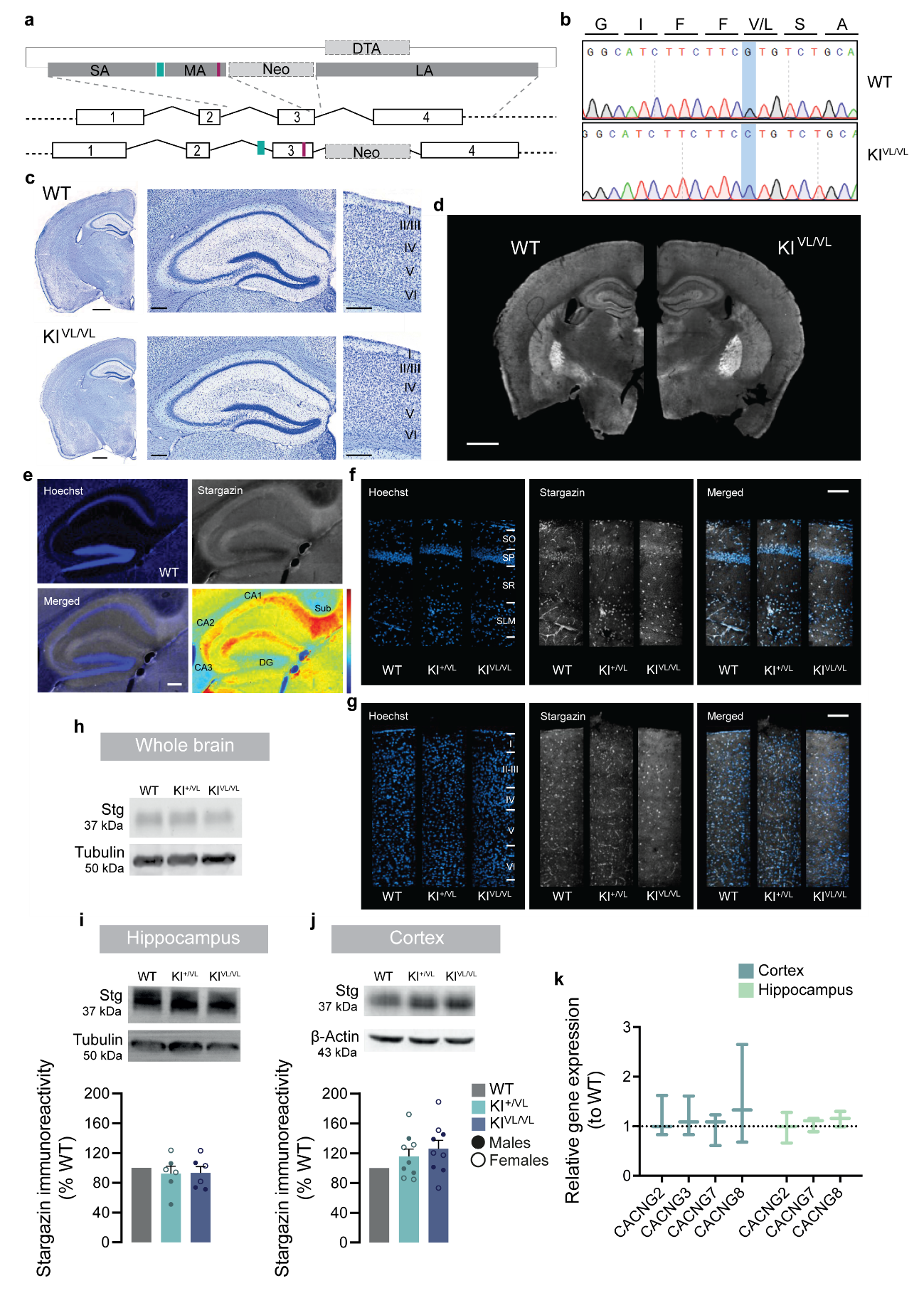


**Figure S4. Stargazin V143L knock-in mice express normal stargazin levels and present no gross brain abnormalities.**

(**a**) Strategy for generating stargazin V143L knock-in mice. A vector containing two selection markers, Neo and DTA, and three homology arms was constructed: the short (SA) and the long arm (LA) allowed homologous recombination with the genomic DNA of mouse embryonic stem (ES) cells, and the middle arm (MA) contained the ID-associated point mutation (V143L) (red bar). A forward primer designed against a synthetic random sequence (non-existing in the mouse genome – green bar), inserted upstream the middle arm, allows genotyping of the animals. (**b**) The directed mutagenesis was confirmed by Sanger sequencing of the third exon in WT and homozygous stargazin KI^VL/VL^ animals. (**c**) Cresyl violet staining of brain slices from WT and stargazin KI^VL/VL^ animals showed no gross differences. Scale bar represents 1000 μm for lower magnification images and 200 μm for magnified images. (**d**) The brain expression pattern of stargazin in WT and stargazin KI^VL/VL^ animals was assessed by immunohistochemistry. Scale bar represents 1000 μm. (**e**) Immunostaining of a sagittal section of a 2-month-old WT mouse and pseudo-colored image of stargazin distribution in the hippocampus. Hot colors indicate higher protein expression levels. Within the hippocampus stargazin is particularly localized in the *stratum lacunosum moleculare* (SLM) and *stratum oriens* (SO) of the CA1 and CA2 regions and more so in the subiculum (Sub). Nuclei were stained with Hoechst 33342. Scale bar represents 200 µm. DG, dentate gyrus. (**f,g**) Representative confocal images of stargazin immunostaining in sagittal slices of 2-month-old WT and stargazin V143L KI mice showing a similar expression pattern in the (**f**) hippocampal CA1 region and in the (g) cortex of stargazin V143L KI animals compared to WT controls. No alterations in lamination of the cortex and hippocampus were observed. Nuclei were stained with Hoechst 33342. Scale bar represents 100 µm. SO, *stratum oriens*; SP, *stratum pyramidale*; SR, *stratum radiatum*; SLM, *stratum lacunosum moleculare*. (**h**) Total stargazin levels in the whole brain, (**i**) hippocampal and (**j**) cortical lysates from WT and stargazin V143L KI animals were evaluated by Western blot. No significant alterations of stargazin levels were observed in (**i**) hippocampal (*p*=0.4844 for KI^+/VL^ and *p*=0.4645 for KI^VL/VL^ animals) or (**j**) cortical lysates (*p*=0.1388 for KI^+/VL^ and *p*=0.0535 for KI^VL/VL^ animals) from stargazin V143L KI mice compared to WT controls. Data are presented as mean ± SEM. One-sample *t*-test to the value of 100%. N = 6 for hippocampal samples and N = 9 for cortical samples for all genotypes. (**k**) Relative gene expression of *Cacng2, Cacng3*, *Cacng7* and *Cacng8* is not altered in the cortex (Kruskal-Wallis test, *p*=0.9034) or hippocampus (Kruskal-Wallis test, *p*=0.7056) of stragazin KI^VL/VL^ animals, in comparison to WT animals. Gene expression values in stragazin KI^VL/VL^ animals are normalized for the expression levels in WT littermates. The expression level of each gene was normalized to the expression of the control gene *Hprt* in the corresponding condition. Data are presented as median and range. N=3 for all conditions.

**
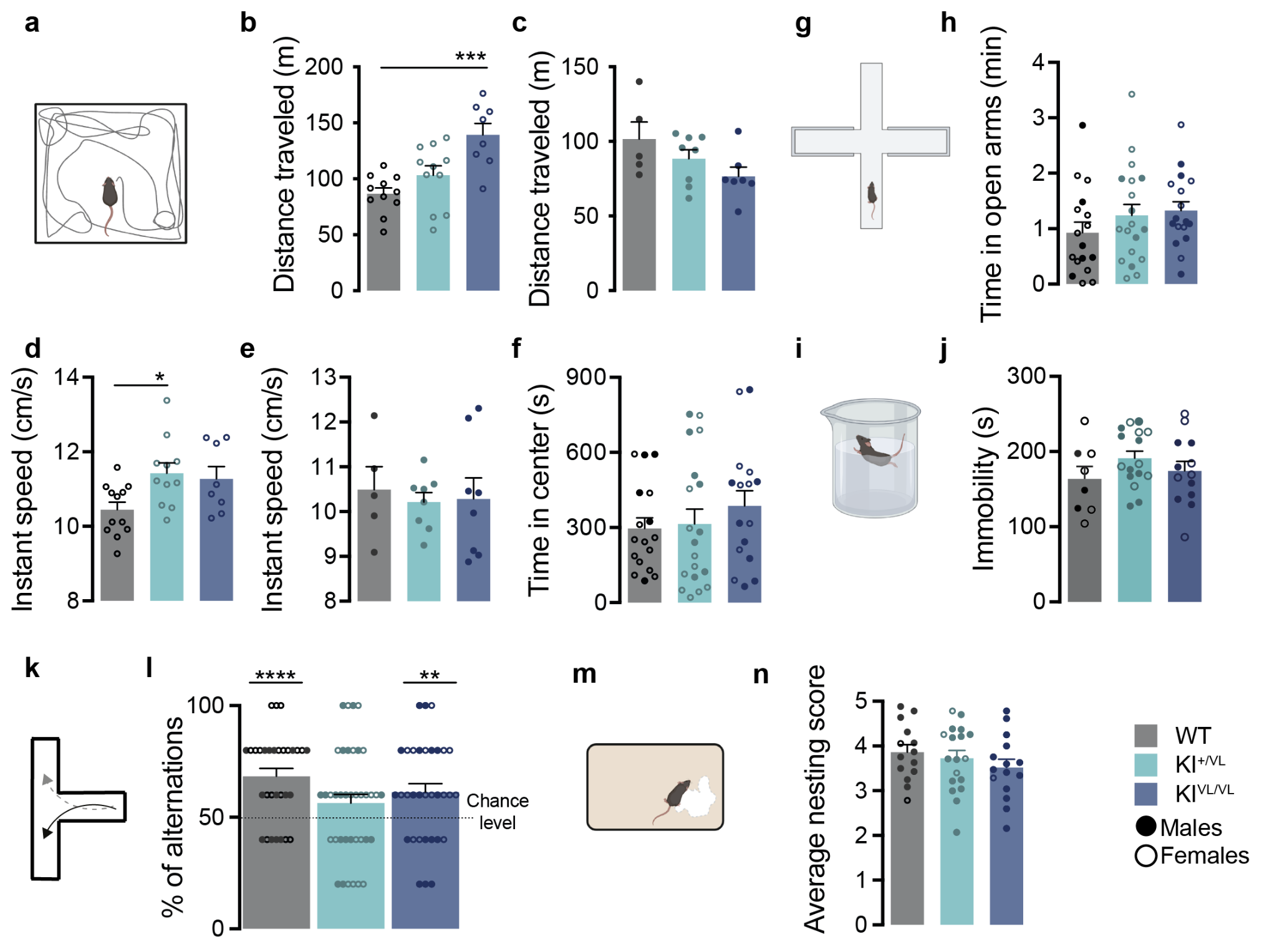
**

**Figure S5. Stargazin V143L KI mice present hyperactivity and working memory deficits.**

(**a**) The locomotor activity was evaluated using the open field test. (**b**) Female stargazin KI^VL/VL^ animals travelled significantly more than WT or heterozygous animals. One-way ANOVA (*p*=0.0004) followed by Dunnet’s multiple comparison tese, ****p*<0.001, N≧8. (**c**) No changes were observed among male animals from different genotypes (one way ANOVA *p*=0.1152, N≧5). (**d**) The instant speed of stargazin KI^+/VL^ female mice was significantly higher than that of WT female mice, but (**e**) did not differ among males from the three genotypes. One-way ANOVA (*p*=0.0213 and *p*=0.8964, respectively), followed by Dunnet’s multiple comparison test, **p<0.05,* N≧8 for females and N≧5 for males. (**f**) Mutant animals did not present changes in the time spent in the center of the open field arena (Kruskall-Wallis test, *p*=0.4988, N≧16), (**g,h**) in the open arms of the elevated plus maze (one-way ANOVA, *p*=0.3055, N≧17 for all genotypes) or (**i,j**) in the immobility time in the forced swimming test (one-way ANOVA, *p*=0.3060, N⋝8 for all genotypes). Together, these data suggest that these animals do not present anxiety-like behaviors. (**k**) Spontaneous alternation was evaluated using the T-maze. (**l**) WT and stargazin KI^VL/VL^ animals alternated significantly more than 50% of times, whereas stargazin KI^+/VL^ animals failed to do so. One-sample Wilcoxon signed-rank test against a value of 50%, *****p*<0.0001, *p*=0.1559, ***p*=0.0097, respectively. N≧31 (males and females) for all genotypes. (**m,n**) In the nesting behavior test, scores given by blind-to-genotype observers did not significantly vary among genotypes (one-way ANOVA, *p*=0.4192). N≧ 14 for all genotypes (males and females). Data are presented as mean ± SEM.


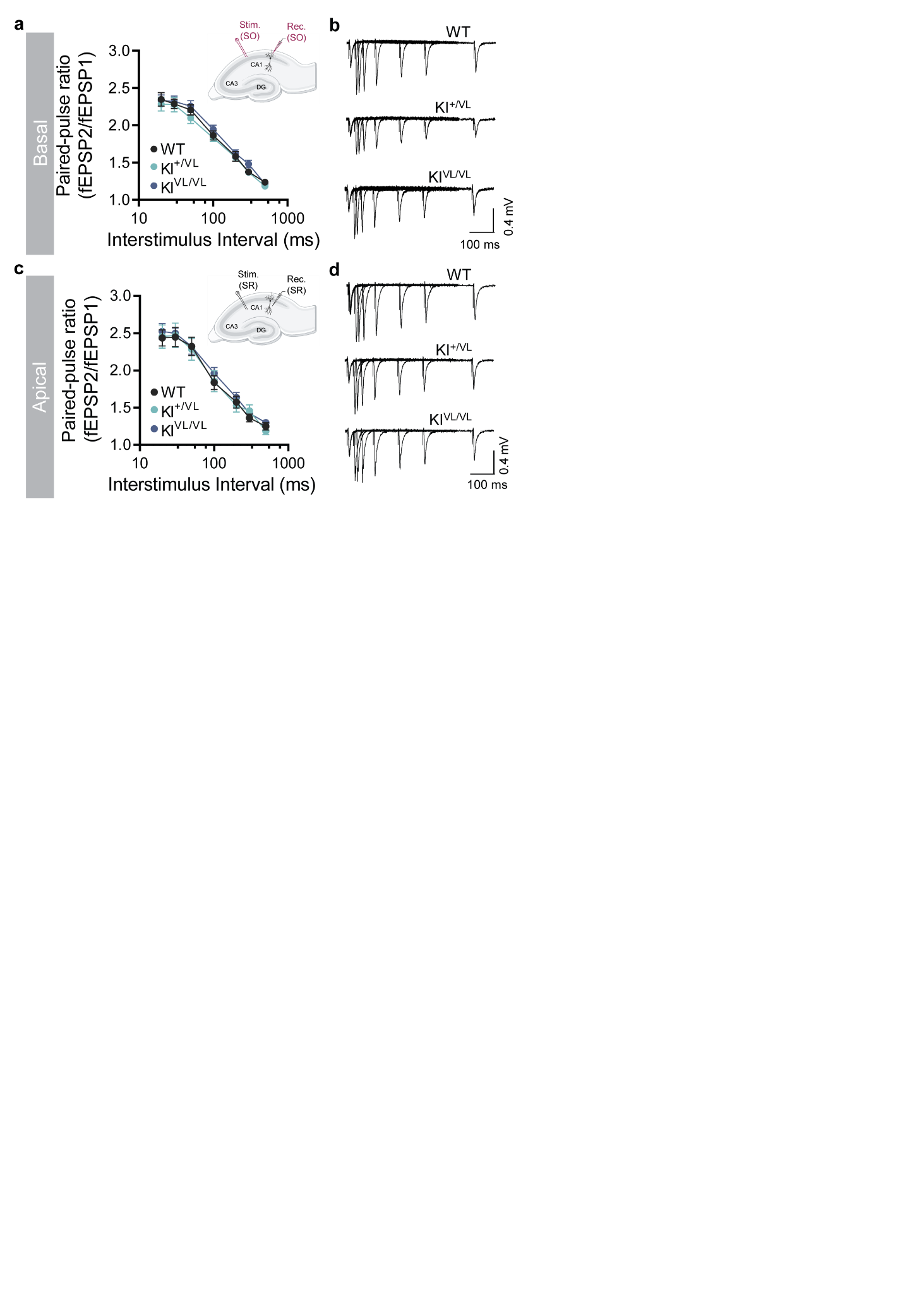


**Figure S6. Short-term plasticity in the CA1 region is not impaired in stargazin V143L KI mice.**

(**a**) Paired-pulse facilitation at basal CA1 dendritic synapses in stargazin V143L Ki mice was not altered [two-way repeated measures ANOVA, *p*=0.9554 (interaction), *p*=0.4088 (genotype), *p*<0.0001 (stimulus intensity)]. The inset shows a schematic representation of electrodes placement for evoked fEPSPs in basal dendritic synapses. Data are presented as means ± SEM. N = 9 slices, N = 5 animals (3 males and 2 females) for WT mice; n = 11 slices, N = 5 animals (2 males and 3 females) for stargazin KI^+/VL^ mice, n = 11 slices, N = 4 animals (2 males and 2 females) for stargazin KI^VL/VL^ mice. (**b**) Representative traces of paired-pulse stimulation evoked fEPSPs in WT and stargazin V143L KI mice when stimulating and recording from the *stratum oriens* (SO). (**c**) Paired-pulse facilitation of apical CA1 dendritic synapses in stargazin V143L mice was not altered [two-way repeated measures ANOVA, *p*=0.9965 (interaction), *p*=0.7827 (genotype), *p*<0.0001 (stimulus intensity)]. The inset shows a schematic representation of electrodes placement for evoked fEPSPs in CA1 apical dendritic synapses. Data are presented as mean ± SEN. N = 8 slices, N = 6 animals (3 males and 3 females) for WT mice; n = 7 slices, N = 5 animals (3 males and 2 females) for stargazin KI^+/VL^ mice; n = 8 slices, N = 5 animals (3 males and 2 females) for stargazin KI^VL/VL^ mice. (**d**) Representative traces of paired-pulse stimulation evoked fEPSPs in WT and stargazin V143L KI mice when stimulating and recording from the *stratum radiatum* (SR).


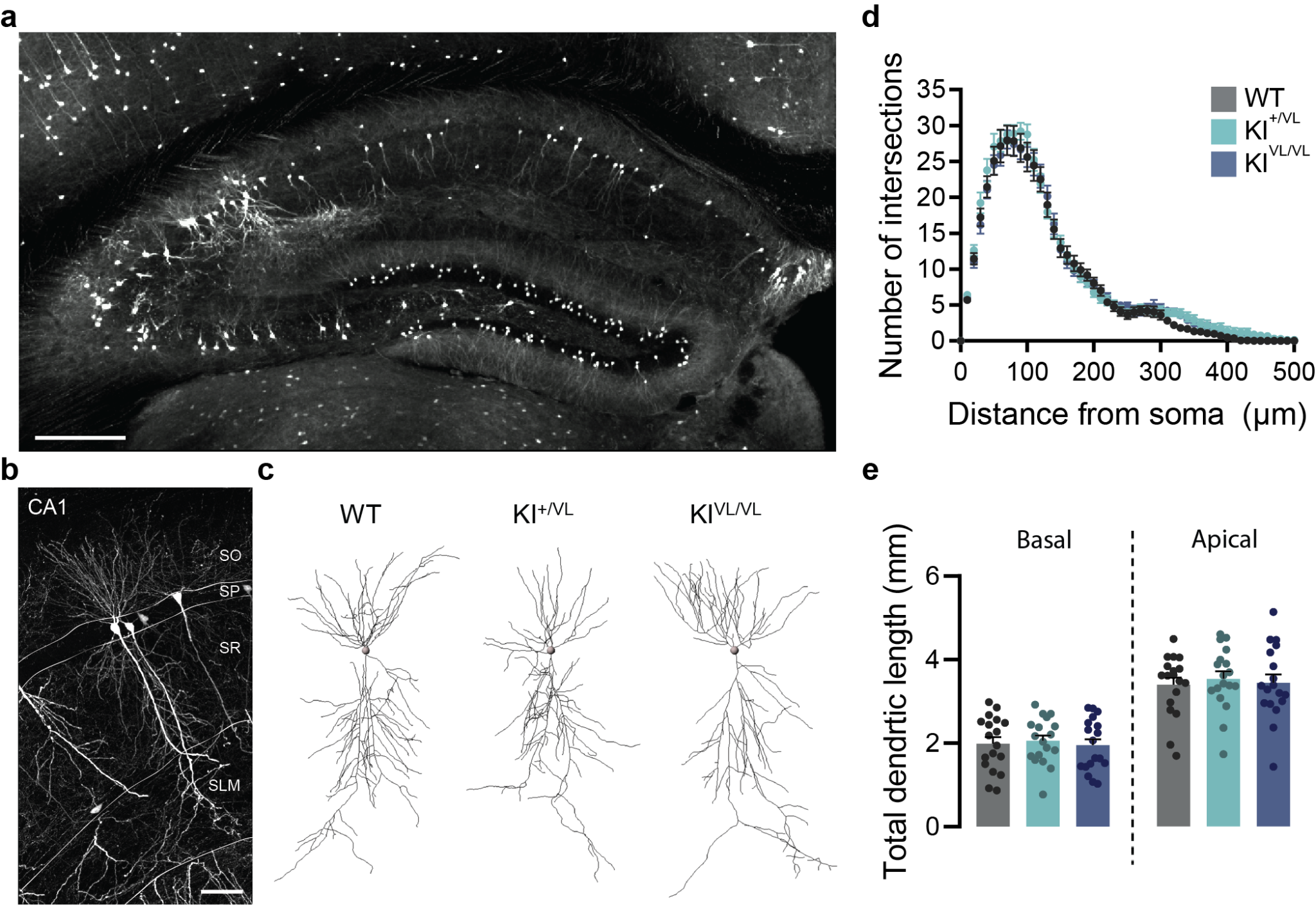


**Figure S7. The morphology of hippocampal CA1 pyramidal neurons is not altered in stargazin V143L KI mice.**

(**a**) Representative image of sparsely labelled hippocampal neurons after tail vein injection with AAV9.hSyn.eGFP. Scale bar represents 300 µm. (**b**) High-magnification representative image showing GFP-labelled neurons in the CA1 region. Scale bar represents 50 µm. (**c**) Representative images of 3D reconstructions of CA1 pyramidal neurons from WT, stargazin KI^+/VL^ and stargazin KI^VL/VL^ mice. (**d**) Sholl analysis showed that there are no significant changes in the dendritic tree architecture of CA1 pyramidal neurons of stargazin V143L mice [two-way repeated measures ANOVA, *p*=0.9987 (interaction), *p*=0.5613 (genotype), *p*<0.0001 (distance from the soma)]. (**e**) The total dendritic length of basal (one-way ANOVA, *p*=0.8673) and apical (one way ANOVA, *p*=0.8523) dendrites are similar for all genotypes. Data are presented as mean ± SEM. n = 18 neurons, N = 3 animals for all genotypes.


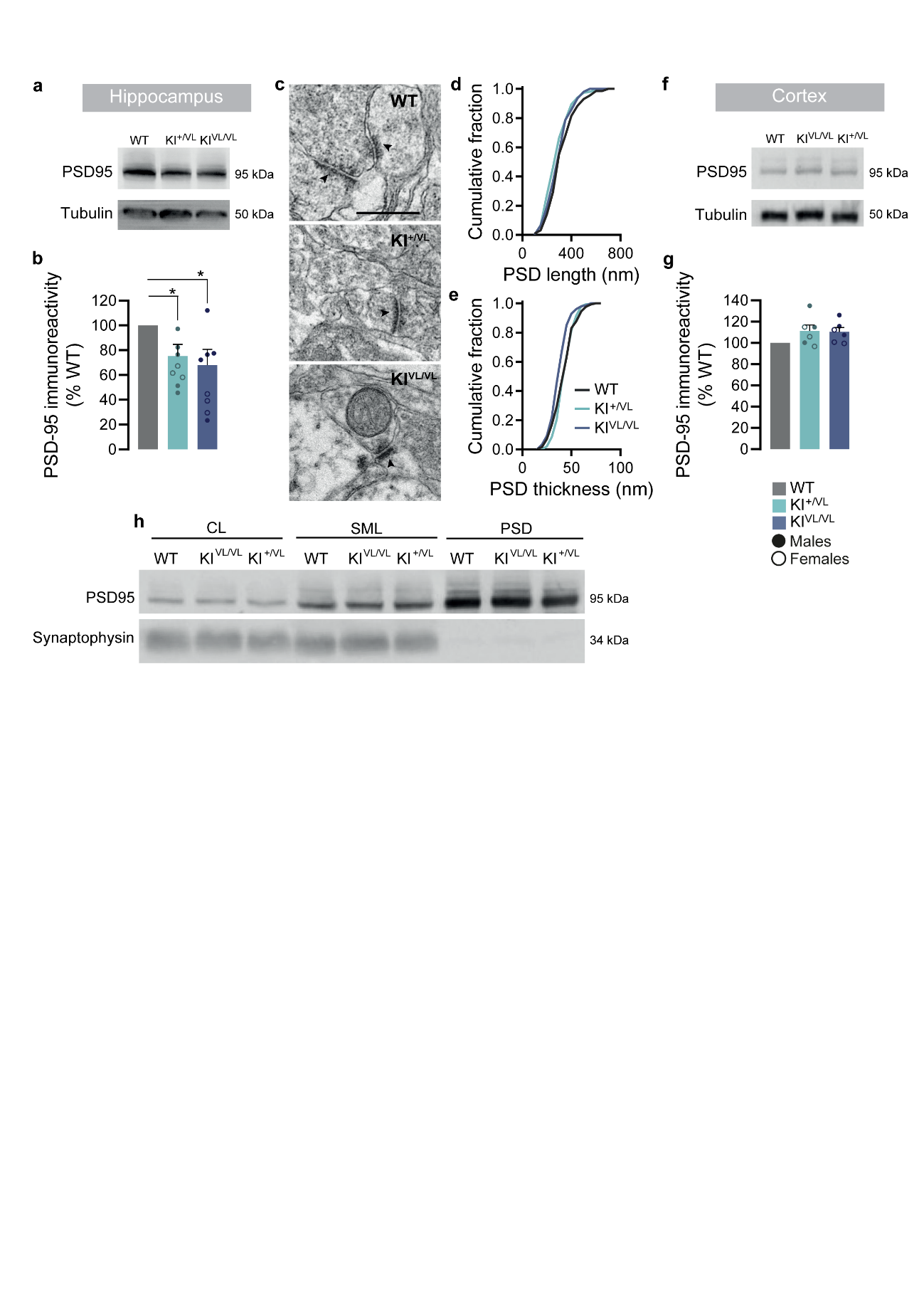


**Figure S8. Stargazin V143L KI mice display alterations in cortical post-synaptic densities ultrastructure.**

(**a,b**) Immunostaining of PSD95 by Western blot showed a significant decrease of its levels in hippocampal samples from stargazin V143L KI mice compared to WT controls. One sample *t*-test to the value of 100%, **p*=0.0313 for KI^+/VL^ and **p*=0.0362 for KI^VL/VL^ animals. The staining for tubulin of hippocampal samples is the same presented in Figure S4i. (**c**) Representative electron transmission microscopy images of cortical synapses from WT, stargazin KI^+/VL^ and KI^VL/VL^ animals and cumulative distribution of post-synaptic density length (**d**) and thickness (**e**). n = 130 PSDs, N = 2 animals for WT; n = 154 PSDs, N = 2 animals for KI^+/VL^ mice; n = 152 PSDs, N = 2 animals for KI^VL/VL^ mice. The arrows indicate the post-synaptic densities. SV, synaptic vesicles. Scale bar represents 500 nm. (**f,g**) Immunostaining of PSD95 by Western blot showed no significant alterations in its levels in cortical samples from stargazin V143L KI mice compared to WT controls (one-sample *t*-test to the value of 100%, *p*=0.1010 for KI^+/VL^ and *p*=0.0519 for KI^VL/VL^ animals. (**h**) Western blot analysis of cellular lysate (CL), synaptic membrane lysate (SML) and post-synaptic densities (PSD) isolated from the brain cortex shows enrichment of PSD95 and absence of synaptophysin labeling in PSDs, confirming efficient isolation of this fraction.

**SUPPLEMENTARY TABLES**

**Table S1.** **Statistical analysis of data.** Details concerning the number of independent experiments, statistical tests used and *p*-values.

| Figure 1 | Panel | Statistical test | *p*-value | Post-hoc test | n | N | Brown-Forsythe test |
| --- | --- | --- | --- | --- | --- | --- | --- |
| Residence time | 1i | Two-tailed Mann-Whitney | *p*<0.0001 | - | 492 | 3 | - |
| Synaptic diffusion coefficient | 1j | Two-tailed Mann-Whitney | *p*=0.0711 | - | 153 | 3 | - |
| Global diffusion coefficient | 1k | Two-tailed Mann-Whitney | *p*=0.0054 | - | 309 | 3 | - |
| GluA synaptic cluster intensity/length | 1m | Kruskal-Wallis | *p*<0.0001 | Dunn's multiple comparison test (*p*-values corrected for multiple comparisons) | ≥53 | 6 | - |
| GluA total surface cluster intensity/length | 1n | Kruskal-Wallis | *p*<0.0001 | Dunn's multiple comparison test (*p*-values corrected for multiple comparisons) | ≥53 | 6 | - |
| Figure 2 | Panel | Statistical test | *p*-value | Post-hoc test | n | N | Brown-Forsythe test |
| ODT | 2b | One-way ANOVA | *p*=0.0412 | Dunnet's multiple comparison test (*p*-values corrected for multiple comparisons) | - | ≥16 | *p*=0.0856 |
|  | 2b | One-sample *t*-test | *p*=0.0130 | - | - | ≥16 | - |
| Fear conditioning | 2d | One-way ANOVA | *p*=0.0076 | Dunnet's multiple comparison test (*p*-values corrected for multiple comparisons) | - | ≥22 | *p*=0.3038 |
| Rotarod | 2f - WT | Ratio paired *t*-test | *p*<0.0001 | - | - | 17 | - |
|  | 2f - Stg^+/VL^ | Ratio paired *t*-test | *p*=0.0009 | - | - | 21 | - |
|  | 2f - Stg^VL/VL^ | Ratio Paired *t*-test | *p*=0.0522 | - | - | 14 | - |
| 3CT | 3h | Two-way ANOVA | *p*=0.4107 (interaction) | Sidak's multiple comparison test (*p*-values corrected for multiple comparisons) | - | ≥17 | - |
|  |  |  | *p*>0.9999 (genotype) |  |  |  |  |
|  |  |  | *p*<0.0001 (S1 vs E) |  |  |  |  |
|  | 3j | Two-way ANOVA | *p*<0.0001 (interaction) | Sidak's multiple comparison test (*p*-values corrected for multiple comparisons) | - | ≥17 | - |
|  |  |  | *p*>0.9999 (genotype) |  |  |  |  |
|  |  |  | *p*<0.0001 (S1 vs S2) |  |  |  |  |
| Figure 3 | Panel | Statistical test | *p*-value | Post-hoc test | n | N | Brown-Forsythe test |
| mEPSCs amplitude | 3b | Kruskal-Wallis | *p*=0.6125 | Dunn's multiple comparison test (*p*-values corrected for multiple comparisons) | ≥21 | ≥6 |  |
| mEPSCs frequency | 3c | Kruskal-Wallis | *p*=0.0003 | Dunn's multiple comparison test (*p*-values corrected for multiple comparisons) | ≥11 | ≥8 |  |
| Fiber vollley amplitude (basal CA1 dendritic synapses) | 3f | Two-way repeated measures ANOVA | *p*=0.7473 (interaction) | - | ≥9 | ≥5 | - |
|  |  |  | *p*=0.8996 (genotype) |  |  |  |  |
|  |  |  | *p*<0.0001 (stimulus intensity) |  |  |  |  |
|  |  |  | *p*<0.0001 (slice) |  |  |  |  |
| fEPSPs slope (basal CA1 dendritic synapses) | 3f | Two-way repeated measures ANOVA | *p*<0.0001 (interaction) | - | ≥9 | ≥5 | - |
|  |  |  | *p*=0.0957 (genotype) |  |  |  |  |
|  |  |  | *p*<0.0001 (stimulus intensity) |  |  |  |  |
|  |  |  | *p*<0.0001 (slice) |  |  |  |  |
| Fiber vollley amplitude (apical CA1 dendritic synapses) | 3g | Two-way repeated measures ANOVA | *p*=0.9996 (interaction) | - | ≥11 | ≥8 | - |
|  |  |  | *p*=0.8860 (genotype) |  |  |  |  |
|  |  |  | *p*<0.0001 (stimulus intensity) |  |  |  |  |
|  |  |  | *p*<0.0001 (slice) |  |  |  |  |
| fEPSPs slope (apical CA1 dendritic synapses) | 3g | Two-way repeated measures ANOVA | *p*=0.7585 (interaction) | - | ≥11 | ≥8 | - |
|  |  |  | *p*=0.7391 (genotype) |  |  |  |  |
|  |  |  | *p*<0.0001 (stimulus intensity) |  |  |  |  |
|  |  |  | *p*<0.0001 (slice) |  |  |  |  |
| LTP (basal CA1 dendritic synapses) | 3h | Two-way repeated measures ANOVA | *p*<0.0001 (interaction) | - | ≥7 | ≥4 | - |
|  |  |  | *p*=0.0030 (genotype) |  |  |  |  |
|  |  |  | *p*<0.0001 (time) |  |  |  |  |
|  |  |  | *p*<0.0001 (slice) |  |  |  |  |
| LTP (apical CA1 dendritic synapses) | 3i | Two-way repeated measures ANOVA | *p*=0.3715 (interaction) | - | ≥7 | ≥5 | - |
|  |  |  | *p*=0.7383 (genotype) |  |  |  |  |
|  |  |  | *p*<0.0001 (time) |  |  |  |  |
|  |  |  | *p*<0.0001 (slice) |  |  |  |  |
| LTP 50-60 min (basal CA1 dendritic synapses) | 3j | One-way ANOVA | *p*<0.0001 | Dunnet's multiple comparison test (*p*-values corrected for multiple comparisons) | ≥7 | ≥4 | *p*=0.7445 |
| LTP 50-60 min (apical CA1 dendritic synapses) | 3k | One-way ANOVA | *p*=0.9267 | - | ≥7 | ≥5 | *p*=0.3261 |
| Figure 4 | Panel | Statistical test | *p*-value | Post-hoc test | n | N | Brown-Forsythe test |
| Spine density (basal CA1 dendritic spines) | 4b | Two-way repeated measures ANOVA | p<0.0001 (interaction) | Dunnet's multiple comparison test (*p*-values corrected for multiple comparisons) | 24 | 3 | - |
|  |  |  | p=0.3924 (genotype) |  |  |  |  |
|  |  |  | p<0.0001 (spine morphology) |  |  |  |  |
|  |  |  | p<0.0001 (dendritic branch) |  |  |  |  |
| Spine density (apical CA1 dendritic spines) | 4d | Two-way repeated measures ANOVA | p=0.3172 (interaction) | - | 24 | 3 | - |
|  |  |  | p=0.3006 (genotype) |  |  |  |  |
|  |  |  | p<0.0001 (spine morphology) |  |  |  |  |
|  |  |  | p<0.0001 (dendritic branch) |  |  |  |  |
| Figure 5 | Panel | Statistical test | *p*-value | Post-hoc test | n | N | Brown-Forsythe test |
| Stg PSD levels CTX | 5b - Stg^+/VL^ | One-sample *t*-test | *p*=0.0217 | - | - | 10 | - |
|  | 5b - Stg^VL/VL^ | One-sample *t*-test | *p*=0.0084 | - | - | 8 | - |
| GluA1 PSD levels CTX | 5d - Stg^+/VL^ | One-sample *t*-test | *p*=0.021 | - | - | 9 | - |
|  | 5d - Stg^VL/VL^ | One-sample *t*-test | *p*=0.2016 | - | - | 7 | - |
| GluA2 PSD levels CTX | 5c - Stg^+/VL^ | One-sample *t*-test | *p*=0.0324 | - | - | 10 | - |
|  | 5c - Stg^VL/VL^ | One-sample *t*-test | *p*=0.2709 | - | - | 7 | - |
| PSD95 PSD levels CTX | 5e - Stg^+/VL^ | One-sample *t*-test | *p*=0.025 | - | - | 6 | - |
|  | 5e - Stg^VL/VL^ | One-sample *t*-test | *p*=0.7638 | - | - | 7 | - |
| Stargazin immunoprecipitation | 5g | One-sample *t*-test | *p*=0.0032 | - | - | 5 | - |
| Figure S4 | Panel | Statistical test | *p*-value | Post-hoc test | n | N | Brown-Forsythe test |
| Stg total levels Hippocampus | S7i - Stg^+/VL^ | One-sample *t*-test | *p*=0.4844 | - | - | 6 | - |
|  | S7i - Stg^VL/VL^ | One-sample *t*-test | *p*=0.4655 | - | - | 6 | - |
| Stg total levels CTX | S7j - Stg^+/VL^ | One-sample *t*-test | *p*=0.1388 | - | - | 9 | - |
| Stg total levels CTX | S7j - Stg^VL/VL^ | One-sample *t*-test | *p*=0.0535 | - | - | 9 | - |
| Figure S5 | Panel | Statistical test | *p*-value | Post-hoc test | n | N | Brown-Forsythe test |
| OFT - distance travelled females | S7b | One-way ANOVA | *p*=0.0004 | Dunnet's multiple comparison test (*p*-values corrected for multiple comparisons) | - | ≥8 | *p*=0.2557 |
| OFT - distance travelled males | S7c | One-way ANOVA | *p*=0.1152 | - | - | ≥5 | *p*=0.5798 |
| OFT - instant speed females | S7d | One-way ANOVA | *p*=0.0213 | Dunnet's multiple comparison test (*p*-values corrected for multiple comparisons) | - | ≥8 | *p*=0.8056 |
| OFT - instant speed males | S7e | One-way ANOVA | *p*=0.8964 | - | - | ≥5 | *p*=0.2344 |
| OFT - time in center | S7f | Kruskal-Wallis | *p*=0.4988 | - | - | ≥16 | - |
| EPM - time in open arms | S7h | One-way ANOVA | *p*=0.3055 | - | - | ≥17 | *p*=0.6041 |
| FST | S7j | One-way ANOVA | *p*=0.3060 | - | - | ≥8 | *p*=0.7761 |
| T-maze | S7l - WT | Wilcoxon signed-rank test | *p*<0.0001 | - | - | 31 | - |
| T-maze | S7l - Stg^+/VL^ | Wilcoxon signed-rank test | *p*=0.1559 | - | - | 38 | - |
| T-maze | S7l - Stg^VL/VL^ | Wilcoxon signed-rank test | *p*=0.0097 | - | - | 33 | - |
| Nesting | S7o | One-way ANOVA | *p*=0.4192 | - | - | ≥14 | *p*=0.8365 |
| Figure S6 | Panel | Statistical test | *p*-value | Post-hoc test | n | N | Brown-Forsythe test |
| Paired-pulse ratio (basal CA1 dendritic synapses) | S6a | Two-way repeated measures ANOVA | *p*=0.9954 (interaction) | - | ≥9 | ≥4 | - |
|  |  |  | *p*=0.4088 (genotype) |  |  |  |  |
|  |  |  | *p*<0.0001 (interstimulus interval) |  |  |  |  |
|  |  |  | *p*<0.0001 (slice) |  |  |  |  |
| Paired-pulse ratio (apical CA1 dendritic synapses) | S6b | Two-way repeated measures ANOVA | *p*=0.9965 (interaction) | - | ≥8 | ≥5 | - |
|  |  |  | *p*=0.7827 (genotype) |  |  |  |  |
|  |  |  | *p*<0.0001 (interstimulus interval) |  |  |  |  |
|  |  |  | *p*<0.0001 (slice) |  |  |  |  |
| Figure S7 | Panel | Statistical test | *p*-value | Post-hoc test | n | N | Brown-Forsythe test |
| Sholl analysis | S7d | Two-way repeated measures ANOVA | *p*=0.9987 (interaction) | - | 18 | 3 | - |
|  |  |  | *p*=0.5613 (genotype) | - |  |  |  |
|  |  |  | *p*<0.0001 (distance from the soma) | - |  |  |  |
|  |  |  | *p*<0.0001 (cell) | - |  |  |  |
| Total dendritic length (basal dendrites) | S7e | One-way ANOVA | *p*=0.8673 | - | 18 | 3 | *p*=0.4294 |
| Total dendritic length (apical dendrites) | S7e | One-way ANOVA | *p*=0.8523 | - | 18 | 3 | *p*=0.7283 |
| Figure S8 | Panel | Statistical test | *p*-value | Post-hoc test | n | N | Brown-Forsythe test |
| PSD-95 total levels Hippocampus | S7b - Stg^+/VL^ | One-sample *t*-test | *p*=0.0313 | - | - | 9 | - |
|  | S7b - Stg^VL/VL^ | One-sample *t*-test | *p*=0.0362 | - | - | 9 | - |
| PSD-95 total levels CTX | S7g - Stg^+/VL^ | One-sample *t*-test | *p*=0.1010 | - | - | 6 | - |
|  | S7g - Stg^VL/VL^ | One-sample *t*-test | *p*=0.0519 | - | - | 6 | - |

**REFERENCES**

1. Sali A, Blundell TL. Comparative protein modelling by satisfaction of spatial restraints. Journal of molecular biology. 1993;234(3):779-815.

2. UniProt C. UniProt: a worldwide hub of protein knowledge. Nucleic acids research. 2019;47(D1):D506-D15.

3. Twomey EC, Yelshanskaya MV, Vassilevski AA, Sobolevsky AI. Mechanisms of Channel Block in Calcium-Permeable AMPA Receptors. Neuron. 2018;99(5):956-68 e4.

4. Sippl MJ. Recognition of errors in three-dimensional structures of proteins. Proteins. 1993;17(4):355-62.

5. Wiederstein M, Sippl MJ. ProSA-web: interactive web service for the recognition of errors in three-dimensional structures of proteins. Nucleic acids research. 2007;35(Web Server issue):W407-10.

6. Wallner B, Elofsson A. Can correct protein models be identified? Protein science : a publication of the Protein Society. 2003;12(5):1073-86.

7. Abraham MJ, Hess B, van der Spoel D, Lindahl E, The-GROMACS-development-team. GROMACS User Manual version 2018.3. GROMACS User Manual version 20183. Groningen2018.

8. Huang J, MacKerell AD, Jr. CHARMM36 all-atom additive protein force field: validation based on comparison to NMR data. Journal of computational chemistry. 2013;34(25):2135-45.

9. Brooks BR, Brooks CL, 3rd, Mackerell AD, Jr., Nilsson L, Petrella RJ, Roux B, et al. CHARMM: the biomolecular simulation program. Journal of computational chemistry. 2009;30(10):1545-614.

10. Wu EL, Cheng X, Jo S, Rui H, Song KC, Davila-Contreras EM, et al. CHARMM-GUI Membrane Builder toward realistic biological membrane simulations. Journal of computational chemistry. 2014;35(27):1997-2004.

11. Berendsen HJC, Postma JPM, Van Gunsteren WF, Di Nola A, Haak JR. Molecular Dynamics with Coupling to an External Bath. J Chem Phys 1984;81(8):3684–90.

12. Darden T, York D, Pedersen L. Particle Mesh Ewald: An N·log(N) Method for Ewald Sums in Large Systems. J Chem Phys 1993;98(12):10089–92.

13. Hess B, Bekker H, Berendsen HJC, Fraaije JGEM. LINCS: A Linear Constraint Solver for Molecular Simulations. J Comput Chem. 1997;18(12):1463–72.

14. Grant BJ, Rodrigues AP, ElSawy KM, McCammon JA, Caves LS. Bio3d: an R package for the comparative analysis of protein structures. Bioinformatics. 2006;22(21):2695-6.

15. Yu H, Dalby PA. A beginner's guide to molecular dynamics simulations and the identification of cross-correlation networks for enzyme engineering. Methods in enzymology. 2020;643:15-49.

16. Munteanu CR, Pimenta AC, Fernandez-Lozano C, Melo A, Cordeiro MN, Moreira IS. Solvent accessible surface area-based hot-spot detection methods for protein-protein and protein-nucleic acid interfaces. Journal of chemical information and modeling. 2015;55(5):1077-86.

17. Miller **BR**, McGee TD, Swails JM, Homeyer N, Gohlke H, Roitberg AE. MMPBSA.py: An Efficient Program for End-State Free Energy Calculations. Journal of chemical theory and computation. 2012;8:3314-21.

18. Valdes-Tresanco MS, Valdes-Tresanco ME, Valiente PA, Moreno E. gmx_MMPBSA: A New Tool to Perform End-State Free Energy Calculations with GROMACS. Journal of chemical theory and computation. 2021;17(10):6281-91.

19. Kaech S, Banker G. Culturing hippocampal neurons. Nature protocols. 2006;1(5):2406-15.

20. Jiang M, Deng L, Chen G. High Ca(2+)-phosphate transfection efficiency enables single neuron gene analysis. Gene Ther. 2004;11(17):1303-11.

21. Opazo P, Labrecque S, Tigaret CM, Frouin A, Wiseman PW, De Koninck P, et al. CaMKII triggers the diffusional trapping of surface AMPARs through phosphorylation of stargazin. Neuron. 2010;67(2):239-52.

22. Groc L, Heine M, Cognet L, Brickley K, Stephenson FA, Lounis B, et al. Differential activity-dependent regulation of the lateral mobilities of AMPA and NMDA receptors. Nat Neurosci. 2004;7(7):695-6.

23. Heyer MP, Feliciano C, Peca J, Feng G. Elucidating Gene Function through Use of Genetically Engineered Mice. Genomics: Essential Methods: John Wiley & Sons, Ltd; 2010. p. 211–48.

24. Deacon RM. Assessing nest building in mice. Nature protocols. 2006;1(3):1117-9.

25. Clements JD, Bekkers JM. Detection of spontaneous synaptic events with an optimally scaled template. Biophysical journal. 1997;73(1):220-9.

26. Edfawy M, Guedes JR, Pereira MI, Laranjo M, Carvalho MJ, Gao X, et al. Abnormal mGluR-mediated synaptic plasticity and autism-like behaviours in Gprasp2 mutant mice. Nat Commun. 2019;10(1):1431.

27. Matt L, Kirk LM, Chenaux G, Speca DJ, Puhger KR, Pride MC, et al. SynDIG4/Prrt1 Is Required for Excitatory Synapse Development and Plasticity Underlying Cognitive Function. Cell Rep. 2018;22(9):2246-53.

28. Fernandes D, Santos SD, Coutinho E, Whitt JL, Beltrao N, Rondao T, et al. Disrupted AMPA Receptor Function upon Genetic- or Antibody-Mediated Loss of Autism-Associated CASPR2. Cereb Cortex. 2019;29(12):4919-31.

29. Zhang H, Zhang C, Vincent J, Zala D, Benstaali C, Sainlos M, et al. Modulation of AMPA receptor surface diffusion restores hippocampal plasticity and memory in Huntington's disease models. Nat Commun. 2018;9(1):4272.
